# The Relationship Between Internet Use, Achievement, and Persistence in Digital Tasks

**DOI:** 10.1002/jad.12503

**Published:** 2025-04-13

**Authors:** Francesca Borgonovi, Elodie Andrieu

**Affiliations:** ^1^ Social Research Institute, Institute of Education University College London London United Kingdom; ^2^ OECD Centre for Skills Organisation for Economic Cooperation and Development Paris France; ^3^ Paris School of Economics Paris France

**Keywords:** accuracy, internet use, performance decline, PISA, secondary‐school students, task persistence

## Abstract

**Introduction:**

As technology progresses, individuals will be increasingly expected to solve digital tasks. At the same time, many worry that a high use of connected devices will reduce young people's ability to perform with accuracy long cognitively challenging tasks online.

**Methods:**

We examine whether 15‐year‐old students' ability to accurately solve cognitively challenging digital tasks—and to maintain accuracy throughout the 2‐h PISA low‐stakes —reflects their frequency of internet use. We do so using data from 153,603 students from 27 countries who participated in the 2018 Programme for International Student Assessment (PISA).

**Results:**

Compared to students with moderate levels of internet use, students who use the internet a lot and those who never use it (or use it very little), have lower baseline levels of accuracy in all key assessment domains (reading, mathematics and science). By contrast, students' use of the internet is not associated with how much accuracy declines over the 2‐h assessment when students are required to solve mathematics and science tasks. In reading, students who use the internet a lot have lower declines in accuracy over the course of the 2‐h assessment compared to students with medium levels of internet use who, in turn, have lower declines than students with low levels of internet use. Internet use is not associated with how carefully students respond to questions in the background questionnaire.

**Conclusions:**

Worries about internet use reducing young people's persistence appear unfounded. At the same time high levels of internet use are associated with low baseline accuracy.

## 1 Introduction

In the past two decades the amount of time young people spend online has increased markedly. As new technologies emerge many worry that high levels of internet use may undermine young people's capacity to sustain attention and broader cognitive development, as well as reshape what it means to be literate (Leu 2000). For example, evidence indicates that since 2009 young people who engage heavily with internet‐connected digital applications have lower levels of academic achievement and lower levels of motivation when performing academic tasks, compared to those with moderate levels of internet use (Vedechkina and Borgonovi 2021; Borgonovi and Pokropek 2021). Young people who spend a substantial amount of time online are also more likely to suffer from problematic internet use, poorer sleep quality, increased fatigue, and to display lower conscientiousness (Sánchez‐Fernández et al. 2023). These factors could negatively influence their overall achievement and their capacity for task persistence. At the same time, other researchers argue that developing the capacity to maintain engagement in lengthy online cognitive tasks can be learnt, and that early exposure and constant access to digital applications are creating a generation of digital natives who can engage with virtual realities and digital technologies productively (Prensky 2001; Kirschner and De Bruyckere 2017).

In this study, we use data on large, representative samples of 15‐year‐old students from 27 countries that participated in the 2018 large‐scale, computer‐based, timed, low‐stakes Programme for International Student Assessment (PISA) test. Although in 2018 as many as 79 countries participated in PISA, 27 countries also opted to administer the Information and Communication Technology (ICT) questionnaire to participating students. Our contribution is twofold. First, we examine if internet use is associated with accuracy in the PISA test. Second, we investigate if internet use is related to declines in accuracy over the course of the 2‐h assessment.

We focus on internet use because online environments are where young people generally access new technologies and tools (Leu 2000), and because empirically this measure is strongly correlated with several distinct indicators of digital use, such as playing videogames, using social media, and engaging with other specific applications. Our primary focus is on reading because reading underpins understanding in other academic subjects, and it is crucial for success in education and beyond (Smith et al. 2000). At the same time, we provide results for mathematics and science to identify the broad implications of internet use.

## 2 Background and Related Literature

In recent years, children have started to access the internet at younger ages, and adolescents have increased the amount of time they spend online (Chaudron et al. 2018; Hooft Graafland 2018). This trend has prompted concerns about potential impacts of internet use on academic achievement and ability to successfully complete long cognitive tasks.

Internet use could have negative effects on how accurately young people solve reading tasks if time spent browsing the internet and using online applications were to displace activities that are known to be strongly and positively associated with the development of reading abilities. Even though children spend some of their time online practicing reading, empirical evidence suggests that reading on screens is associated with poorer outcomes compared to reading on paper, even when outcomes are measured using digital assessments (Ackerman and Goldsmith 2011; Daniel and Woody 2013). At the same time, internet use could yield cognitive benefits that are specific to online reading comprehension (Coiro 2011), for instance by promoting informal exploratory learning and enhancing problem‐solving skills (Feng and Spence 2018). In line with prior empirical evidence (Borgonovi and Pokropek 2021), we expect moderate internet users to display the highest levels of accuracy in the PISA reading test, since they can benefit from the learning opportunities internet‐related activities offer without entirely displacing other valuable activities.

Adolescents with different patterns of internet use may also differ in their ability to maintain accuracy over the course of a long cognitive test, a factor that has meaningful economic and social consequences and that shapes inequalities and the accumulation of social advantage (Radl and Miller 2021). During adolescence, the capacity to maintain high levels of accuracy over long cognitive tests is associated with grades and educational attainment, and among young adults, it correlates with income and more effective education‐to‐work transitions, both directly and indirectly through its influence on educational outcomes (Borgonovi et al. 2021; Andersson and Bergman 2011).

Internet use could influence young people's capacity to maintain high levels of accuracy over long cognitive tests by shaping how tired adolescents are, their executive functions, attention, and motivation while performing digital tasks. Adolescents who spend a lot of time online often have poorer sleep habits (Alimoradi et al. 2019), and sleep deprivation worsens young people's performance on academic exams (Okano et al. 2019). Furthermore, adolescents who are heavy users of the internet often practice digital multitasking (Ettinger and Cohen 2020), which may interfere with the development of attention networks and executive functions, leading to attention difficulties and frequent task switching (Levine et al. 2007). However, the computer‐based, problem‐oriented nature of the PISA test could align well with interests and preferences of heavy internet users, potentially enhancing their motivation during the completion of long cognitive tests. These competing factors lead us to have no strong a priori hypotheses regarding the direction of the association between internet use and young people's capacity to maintain high levels of accuracy over the PISA test.

Methodologically, we build on the literature on item position effects. We consider more pronounced negative position effects (i.e. lower accuracy on items that are placed towards the end rather than the beginning of the test) to indicate lower capacity to maintain high levels of accuracy and, vice versa, we consider less pronounced negative position effects as indicative of greater capacity to maintain high levels of accuracy. In line with previous empirical work, we exploit the rotation design implemented in PISA to estimate how accuracy varies over the course of the PISA test (Borgonovi and Biecek 2016; Borghans and Schils 2012; Zamarro et al. 2019; Debeer et al. 2014; Wu et al. 2019). Because prior research on item position effects based on analyses of PISA revealed that declines in accuracy are generally larger in reading than in science and mathematics (Borgonovi and Biecek 2016), we also estimate associations between internet use and changes in accuracy as a function of item position in mathematics and science. Finally, we consider whether our results reflect broader behavioural tendencies using measures of the behaviour adopted by students as they completed the main background questionnaire. Such behaviours have been used in the literature as indicators of personality traits and motivation (Borgonovi et al. 2023; Soland et al. 2019).

## 3 Materials and Methods

### 3.1 Programme for International Student Assessment

PISA is a triennial low‐stakes international assessment. The first edition was conducted in 2000. The PISA target population comprises 15‐year‐olds attending lower or upper secondary schools and our data come from the public‐use 2018 edition of PISA, available at http://www.oecd.org/pisa/data. We exclude from our study students who reported that either they or their parents were not born in the country where they sat the PISA test. These students may differ in accuracy and task persistence because of additional fatigue related to language difficulties (OECD 2018) or other factors that we are not able to control for.

After the 2‐h test, participating students had a short break before completing a core questionnaire. Additionally, in 27 countries, they were administered an ICT questionnaire containing questions on internet use and broader use of ICT technologies. All instruments were administered on computers. The key PISA assessment domains are reading, mathematics and science and in 2018 reading was the main assessment.

### 3.2 The Pisa Assessment Design

In 2018, a multistage adaptive testing design was introduced for reading (Yamamoto et al. 2019). Whereas mathematics and science materials were organised into blocks of items designed to take approximately 30 min to complete, reading materials were organised into three stages – core, first stage and second stage ‐ designed to take around 60 min to complete. The core stage did not include any adaptive elements, whereas stage 1 and stage 2 were adaptive. Using information on item difficulty and accuracy in automatically scored items in the core stage, students were categorised into high, medium, and low ability groups. High‐ability students were assigned with a 90% probability items of higher average difficulty in the first stage, and with a 10% probability items of lower average difficulty. Conversely, low‐ability students were more likely to receive easier items and less likely to receive harder items. Medium‐ability students had a 50% probability of being assigned items of higher average difficulty and 50% of being assigned items of lower average difficulty. Allocation in the second stage followed the same principle, based on item difficulty and accuracy data from all previously automatically scored items.

### 3.3 Variables

#### 3.3.1 Outcome Variable

##### 3.3.1.1 Accuracy

Accuracy is dichotomous and takes value 1 when students provided a correct or partially correct response and 0 if students responded incorrectly. Following Wu et al. (2019) we coded non‐reached items as missing and non‐attempted as an incorrect answer. On average across our sample students had an accuracy rate of 61%. One of the main advantages of using data from PISA in the analysis is that the PISA assessment is developed by international experts in collaboration with representatives of education authorities from member countries. This collaboration ensures the standardised nature of the instruments and the implementation of rigorous technical standards to guarantee comparability. These standards include a field trial that is conducted before the main study administration to validate instruments and administration protocols (OECD 2019a). In PISA the same assessment items are administered across all participating countries, and great care is taken to ensure that translations of items in different languages or other administration conditions do not influence the ease with which students can solve items in different contexts.

In 2018, a total of 244 reading items, 82 mathematics items, and 115 science items were administered. However, each student received only a subset of the full item pool. Test items were developed not only to assess proficiency across a range of cognitive processes and content areas, but also to incorporate a diverse array of stimuli and response formats. Additionally, some items were scored by computer, while others were scored by human coders. A comprehensive description of the test items in the PISA assessment is available in Annex A of the PISA 2018 Technical Report (OECD 2019d).

#### 3.3.2 Key Independent Variables

##### 3.3.2.1 Item Position

We determined the position of each reading, science, and mathematics item within the test administered to each student. Our indicator of item position is the percentile rank in the ordering of questions for each individual student, with lower numbers indicating items presented towards the beginning of the test and higher numbers indicating items presented towards the end. This approach accounts for the fact that different students received a different number of items in the test and received them in different positions.

##### 3.3.2.2 Internet Use

We measure the level of internet use using the optional ICT questionnaire administered to students immediately after they had completed the assessment part of the PISA study. Students were asked, “During a typical weekday, for how long do you use the internet outside of school?” and could select one of the following response categories: ‘no time’, ‘1 to 30 min per day’, ‘31 to 60 min per day’, ‘between 1 h and 2 h per day’, ‘between 2 h and 4 h per day’, ‘between 4 h and 6 h per day’, and ‘more than 6 h per day’. The questionnaire did not specify a precise time frame students should consider when evaluating their behaviour on a typical weekday but, rather, were asked to provide a global evaluation of their current use as 15‐year‐old students.

To distinguish between students with low, medium, and high levels of use, we categorise students into three groups: those who report not using the internet or using it for less than half an hour per day (low use), those who report using the internet for between half an hour and 4 h per day (medium use), those who report using the internet for over 4 h (high use). Around 6% of students were in the low internet use category, 54% were in the medium use category and 40% were in the high use category. In all models, students with medium levels of use serve as the reference category, with two dichotomous indicators added for low and high use. The measure of internet use used in the analysis is strongly correlated with indicators available in PISA 2018 on the specific activities students engage in – such as participating in social networks, playing video games, downloading or uploading music, films or games or software – and with frequency of use over week‐ends. The classification of students into three groups of internet use based on the amount of time students report using the internet for is aligned with OECD's own PISA reports and policy documents (OECD 2019b). The specific thresholds adopted in the analysis are driven by empirical evidence on the emergence of adverse health effects associated with internet use (Berchtold et al. 2018). At the same time, results obtained using different categorisations of internet use – such as using different thresholds to assign students into the three categories, using a different number of categories, considering internet use over weekends, or cumulative use over week and week‐end days – are consistent with those reported, although differences between groups may become more or less pronounced and precisely estimated depending on the exact specification.

We opted to display results for the three categories of internet use because using a dichotomous variable would have prevented us to examine potential nonlinear associations and this approach would have oversimplified the relationship between internet use and the outcomes of interest. Additionally, we wanted a parsimonious model and refrained from introducing too many categories, as this would have complicated the interpretation of the models, particularly given the complexity introduced by interaction terms. Furthermore, we chose not to employ a continuous indicator for internet use to avoid implying precise timing, to maintain the integrity of the category distinctions provided in the data set, and to prevent assigning a specific value to the highest category—‘more than 6 h per day’.

### 3.4 Controls

We control for students' socio‐economic status using the PISA index of economic, social, and cultural status (ESCS), an index based on students' responses on their family background including parental education, occupation and home possessions (Avvisati 2020). Socioeconomic status may shape both the probability of performing well on the test and internet use. For similar reasons, we control for age and gender. Girls serve as the reference category and age is treated as a continuous variable ranging from 15 years and 3 months and 16 years and 2 months. The sample was balanced with 50% female students in the sample and the average age is 15 years and 9 months.

Because of evidence that students' response behaviour in the PISA test differs depending on the ease with which they read (Avvisati and Borgonovi 2023), we included an indicator of reading fluency as a control in some specifications. This indicator corresponds to the within‐country percentile distribution of the total time students took to read (and understand) 22 sentences (reading fluency items). The fastest students were assigned a value of 1 (indicating higher fluency) and the slowest student were assigned a value of 100 (indicating lower fluency).

For each item, we included an indicator of the amount of time students spent solving the item and its squared value. This indicator represents the decile within the country‐specific distribution, based on the number of milliseconds a student spent solving the item relative to other students who were assigned to the same item in the same country (OECD 2019c). Using deciles allow us to take into account differences in item length, linguistic variations, and other country‐specific factors that may lead students in different countries to spend a different amount of time on an item.

### 3.5 Questionnaire Response Behaviours

The item nonresponse indicator was developed using students' responses in the main background questionnaire (questions in the ICT questionnaire were not used in the calculation) and represents the share of multiple‐choice questions that students left unanswered out of all multiple‐choice questions. The item nonresponse rate indicator is defined as:

$$\mathrm{Item non}-\mathrm{response}=\frac{\mathrm{Number of applicable items left blank}}{\mathrm{Number of applicable items}}$$

The inconsistency indicator is based on item‐rest correlations, i.e. correlations between an item score and the overall scale score (also called rest score). It measures if students answer a specific item in a way that is unpredictable based on the answers to other items within the same Likert‐type scale. First, we adjusted answers of reverse‐coded items (such as negatively phrased items in positively phrased scales). For each item, we regressed the item response on the average of answers in the remaining items belonging to the scale. Then, we run regressions separately for each country in our sample to account for the fact that the internal consistency of scales might differ across countries and considered only respondents who had non‐missing responses for at least 3 items in a scale. For each item, we fit the following linear model:

$$Y_{\mathrm{ijs}}=\beta_{0}+\beta_{1}\bar{X_{\mathrm{is},-j}}+\eta_{\mathrm{ijs}}$$

*Y*_*ijs*_ represents the answer of student *i* to item *s* in scale *j* and $\bar{X_{\mathrm{is},-j}}$ represents the average response on all remaining items in the scale, *β*_*0*_ represents a constant and *η*_*ijs*_ is an item‐specific, scale‐specific and student‐specific error term representing the degree to which student *i* gave an unpredictable answer to item *s* in scale *j*.

Finally, the non‐differentiation indicator reflects the extent to which students consistently select the same response across a set of similar and related Likert‐type items within a scale. To compute a non‐differentiation metric for each student, we compute the percentage of item sets in which they did not differentiate their answers out of all valid items sets.

$$\mathrm{Non}-\mathrm{differentiation}=\frac{\mathrm{Number of applicable item sets without differentiation}}{\mathrm{Number of applicable item sets}}$$

For a detailed description of how the indicators were constructed see (Borgonovi et al. 2023). All indices were standardised to have a mean of zero and a standard deviation of one across the pooled sample, with higher values indicating less conscientious responses.

### 3.6 Methodological Framework

To estimate the association between internet use and accuracy and how accuracy varies as a function of item position we used a generalized linear mixed approach (GLMM) extending the multidimensional IRT (MIRT) models proposed by De Boeck and Wilson (2004), Debeer et al. (2014) and Weirich et al. (2017). For each country with available data, we fitted the following country‐specific model:

$$\mathrm{logit}\left[Y_{i,p,s,k}=1\right]=\theta_{s}+\theta_{p,s}-\beta_{i}+\gamma_{z}{\mathrm{INTERNET}}_{p}+B_{x}X_{p}+\left(\gamma +\delta_{s}+\delta_{p,s}\right)\left(k_{p,i}-1\right)$$

Where: $Y_{i,p,s,k}$ represents the response of student *p* in school *s*, to reading item *i* in position *k*; $k_{p,i}$ represents the position of item *i* for student *p*; γ represents the general effect (fixed) of the position of items across students; $\delta_{s}+\delta_{p,s}$ represent school and person random effects capturing the school and individual deviation from the average; $\theta_{s}+\theta_{p,s}$ represent the general level of accuracy on the overall assessment of students in school *s* and of student *p* in school *s* $\text{and}\;B_{i}$ represents the difficulty of item i; $X_{p}$ is a vector with student characteristics (controls), ${\mathrm{INTERNET}}_{p}$ is a vector of two dichotomous indicators of whether the student *p* has low or high rather than medium levels of internet use and $\gamma_{z}$ represents the change in accuracy that is associated with low or high levels of internet use rather than medium levels of use.

We augmented this model to examine if the efficiency with which students respond to test items varies as a function of the position of these items. This was achieved by introducing interaction effects between the time students spend responding to an item and the item's position. Additionally, we incorporated interactions between the two internet use indicators and the position of the item within the test. These interactions were included both before and after controlling for the amount of time students spend responding to each item, the internet use indicators, the response time per item indicators and all interaction terms in the model.

Because the adaptive design influences the level of difficulty of test items students are required to solve at different stages in the reading test, it could have an influence on the estimation of declines in accuracy. We tackled this in two ways. First, we excluded students who were mis‐routed (i.e. those allocated a higher difficulty item set despite low accuracy, or to a lower difficulty item set despite high achievement) from all analyses, because misrouting may have interfered with motivation. The total number of mis‐routed students was 25,170. Second, we consistently controlled for item difficulty to ensure that our estimates reflect differences in accuracy for items of similar difficulty^1^.

To estimate the association between internet use and questionnaire response behaviours we fit country specific linear regression models, taking into account the complex survey design of PISA by using balanced repeated replication weights. All analyses were conducted using the R package lme4 (Bates et al. 2015; R Core Team 2014).

Our final sample at the student level differs across models, depending on whether we examine accuracy in reading or mathematics/science, because different students were allocated different testing material. For reading our final sample consists of 128,433 students with complete information on all key independent variables and who were not misrouted during the reading test. For mathematics/science our final sample consists of 153,603 students with complete information on all key independent variables. For the behavioural indicators it consists of 151,092 students with complete information on all key independent variables. Because our analysis is conducted at the item level, the number of observations reflects the number of items administered to participating students. This ranges between 4,325,006 observations in models 1a and 2a in which we estimate reading accuracy to 3,426,239 observations in model 3 in which we estimate accuracy in mathematics and science. The slightly lower number of observations in models 1b and 2b compared to models 1a and 2a reflects the fact that for some items, and for some students, timing data was not recorded properly because of technical malfunctions. Table S1 in the Appendix provides summary statistics and sample composition.

## 4 Results

### 4.1 Internet Use, Accuracy, and Declines In Accuracy

**Figure 1:**
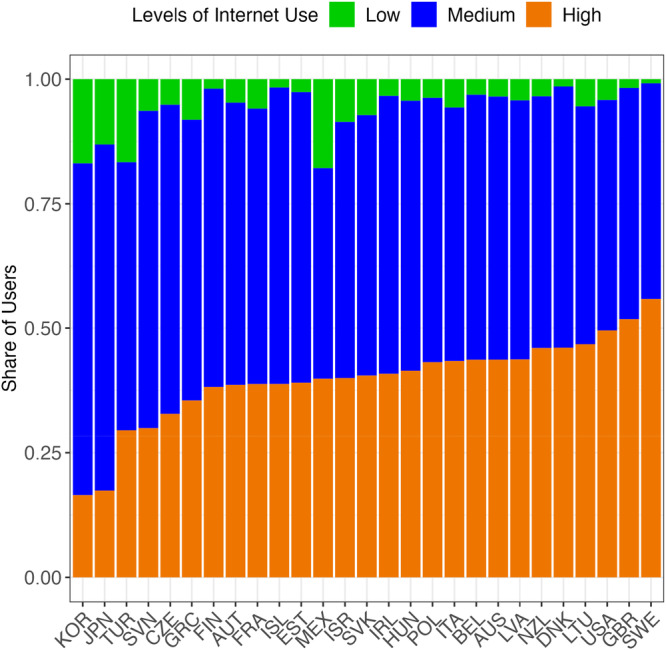
Share of 15‐year‐old students, by level of ICT use and by country.

Figure 1 presents information by country on the level of internet use of 15‐year‐old students. On average across the 27 countries with available data 6% of 15‐year‐old students were in the low level of internet use group, 54% in the medium level of internet use group and 40% were in the high level of internet use group.

In Table 1 we present results in which we estimate the likelihood that a student will respond correctly to a test item, how this varies depending on the position of the test item and their level of internet use. Because unstandardised logistic regression coefficients can be difficult to interpret, to aid interpretability we report and discuss odds ratios (ORs).

**Table 1:** The association between internet use and accuracy in reading, mathematics and science, country average.

	Reading								Mathematics and Science	
	Model 1a		Model 1b		Model 2a		Model 2b		Model 3	
Variables	B	ORs	B	ORs	B	ORs	B	ORs	B	ORs
Constant	2.52***		1.31***		2.54***		1.33***		−4.12***	
	(0.03)		(0.04)		(0.03)		(0.04)		(0.04)	
Girl	0.21***	1.23	0.19***	1.21	0.21***	1.23	0.19***	1.21	−0.17***	0.84
	(0.01)		(0.01)		(0.01)		(0.01)		(0.01)	
Age	0.18***	1.20	0.17***	1.19	0.18***	1.20	0.17***	1.19	0.15***	1.16
	(0.01)		(0.01)		(0.01)		(0.01)		(0.01)	
ESCS Index	0.24***	1.27	0.24***	1.27	0.24***	1.27	0.24***	1.27	0.26***	1.30
	(0.00)		(0.00)		(0.00)		(0.00)		(0.00)	
Reading Fluency	−0.01***	0.99	−0.01***	0.99	−0.01***	0.99	−0.01***	0.99	−0.01***	0.99
	(0.00)		(0.00)		(0.00)		(0.00)		(0.00)	
High Internet User (vs. Medium)	−0.19***	0.83	−0.18***	0.84	−0.24***	0.79	−0.23***	0.79	−0.18***	0.84
	(0.01)		(0.01)		(0.01)		(0.01)		(0.01)	
Low Internet User (vs. Medium)	−0.45***	0.64	−0.43***	0.65	−0.39***	0.68	−0.39***	0.68	−0.42***	0.66
	(0.02)		(0.02)		(0.03)		(0.03)		(0.03)	
Item position (percentile)	−1.14***	0.32	−1.92***	0.15	−1.17***	0.31	−1.96***	0.14	−1.13***	0.32
	(0.01)		(0.04)		(0.01)		(0.04)		(0.04)	
Time on task (decile) ±			0.20***				0.20***		0.23***	
			(0.004)				(0.004)		(0.005)	
Time on task squared (decile) ±			−0.02***				−0.02***		−0.02***	
			(0.00)				(0.00)		(0.00)	
High Internet User (vs. Medium) × Item position (percentile)					0.15***	1.16	0.16***	1.17	0.01	1.01
					(0.02)		(0.02)		(0.02)	
High Internet User (vs. Medium) × Item position (percentile)					−0.33***	0.72	−0.30***	0.74	0.06	1.06
					(0.04)		(0.04)		(0.05)	
Item position (percentile) × Time on task (decile) ±			0.18***				0.18***		0.16***	
			(0.01)				(0.01)		(0.01)	
Item position (percentile) × Time on task squared (decile) ±			−0.01***				−0.01***		−0.01***	
			(0.00)				(0.00)		(0.00)	
Question Fixed Effects	Yes		Yes		Yes		Yes		YES	
*N* (Students × items)	4325006		4097907		4325006		4097907		3426239	

*Note:* The table reports the average unstandardised logistic regression coefficients (B) of equally‐weighted country‐specific estimates. Standard errors SE(B) are reported in parenthesis. Odds Ratios (ORs) are reported next to the logistic regression coefficients ± No Odds Ratios are reported for time on task and its squared term because the relationship between time on task and accuracy is considered to be nonlinear and therefore ORs value depend on the specific value of time on task upon which the change is considered. ESCS Index refers to the PISA Index of Economic, Social and Cultural Status. *N* refers to the number of students*number of items. ** *p* < 5%;

*** *p* < 1%. Country specific estimates can be found in the Supporting Information Annex.

In Model 1a we estimate how accuracy varies depending on students' use of the internet and the position of a specific test item when controlling for key individual level characteristics. In Model 1b we add controls for the time students spent on specific items and how time spent varies as a function of item position to control for the fact that different types of students: students with different levels of internet use may invest a different amount of time trying to solve test items and this would shape their accuracy. When controlling for time, the accuracy indicator reflects how well students respond given the amount of time they spend solving an item. Models 1a and 1b reveal that students who are heavy users of the internet and those who have low levels of use have lower baseline levels of accuracy compared to students who have medium levels of internet use, i.e. they are less likely to respond correctly to a test item when this is placed at the start of the test. For example, results from Model 1b suggest that students with high levels of internet use have odds of giving a correct answer that are about 16% lower than students with medium levels of internet use (*OR* = 0.84; *B* = −0.18; *p* ≤ 0.01). Similarly, students with low levels of internet use have odds of giving a correct answer that are about 35% lower than those of students with medium levels of use (*OR* = 0.65; *B* = −0.43; *p* ≤ 0.01). Table 1 suggests that on average, across countries in our sample, students' level of accuracy varies as a function of the position an item had in the PISA test: the item position variable is negative across all model specifications. For example, results in Model 1a indicate that when test items are placed at the end of the test students have odds of giving a correct answer that are about 68% lower than when they are placed at the start of the test (*OR* = 0.32; *B* = −1.14; *p* ≤ 0.01).

In Models 2a and 2b we replicate analyses reported in Models 1a and 1b but, crucially, we add interaction terms between students' levels of internet use and item position (Model 2a) and between levels of internet use and time on task (Model 2b). These results allow us to consider whether the decline in accuracy varies as a function of internet use and if the association between time spent on items and accuracy varies depending on students' level of internet use. Whereas there is an inverted U relationship between internet use and the likelihood that students will respond correctly to a test question at the start of the test, Models 2a and 2b reveal that the decline in the likelihood that students will respond correctly to a test item that is towards the end rather than the start of the test is linear. Such decline is highest among students with low levels of internet use and is lowest among students with high levels of use: students in the high use group are those with the highest task persistence. Results presented in Model 2a (without controls for time on task) reveal that, on average and holding other factors constant, students with medium levels of internet use have odds of giving a correct answer that are about 69% lower when a question is at the very end of the test than when the same question is placed at the very start (*OR* = 0.31; *B* = −1.17; *p* ≤ 0.01). By contrast, students with high levels of internet use have odds of giving a correct answer that are about 36% lower when a question is at the end of the test than when the same question is at the start (*OR* = 0.36; *B* = −1.17 + 0.15; *p* ≤ 0.01) whereas among students who have low levels of use, the odds are about 78% lower (*OR* = 0.22; *B* = −1.17 − 0.33; *p* ≤ 0.01). Model 2b also indicates that heavy users of the internet decline less in accuracy given a specific amount of time dedicated to test questions depending on the position such question takes. Country specific results on declines in accuracy as a function of internet use are available in Supplementary Online Annex Figure S1. Results suggest that in the majority of countries the decline in accuracy is less pronounced among students who report high levels of internet use than among students with medium levels of use and the difference between the two groups is statistically significant. Furthermore, in 12 countries the decline in accuracy is more pronounced among students who report low levels of internet use than among students who have medium levels of internet use and the difference between the two groups of students is statistically significant. We did not identify a correlation between differences across countries in the association between of internet use and decline in accuracy and country‐specific prevalence of internet use.

### 4.2 Results for Mathematics and Science

Model 3 in Table 1 provides results on the association between internet use and accuracy in mathematics and science. The two domains are considered jointly because a small number of mathematics and science items were administered in 2018. Students in the middle level of internet use group have higher accuracy than students who have high and low levels of use of the internet. Results from Model 3 in fact suggest that students with high levels of internet use have odds of giving a correct answer to mathematics and science items that are about 16% lower than students with medium levels of internet use (*OR* = 0.84; *B* = −0.18; *p* ≤ 0.01). Similarly, students with low levels of internet use have odds of giving a correct answer that are about 34% lower than those of students with medium levels of use (*OR* = 0.66; *B* = −0.42; *p* ≤ 0.01). Furthermore, students on average have odds of responding correctly to mathematics and science test items that are about 16% lower when those items are placed at the end of the assessment than when they are placed at the start (*OR* = 0.84; *BB* = −0.18; *p* ≤ 0.01). However, and contrary to results presented for reading, estimates presented in Model 3 do not reveal differences across students with low, medium, and high levels of internet use in how much accuracy declines as a function of item position: interaction terms between high/low levels of internet use and item position are quantitatively small (i.e. very close to 1) and are not statistically significant.

### 4.3 Internet Use and Questionnaire Response Behaviours

Table 2 indicates that students who have high and low levels of use of the internet do not differ from students with medium levels of use of the internet in terms of item nonresponse and non‐differentiation. In other words, students with different levels of internet use have similar rates of multiple‐choice questions with missing responses and they are equally likely to provide non‐differentiated responses in the background questionnaire. The only indicator for which we identify statistically significant differences is non‐consistency and the effect is not large: being in the high rather than the medium level of use of the internet group is associated with a 12% of a SD difference in the non‐consistency indicator and being in the low rather than the medium level of use of the internet group is associated with a 32% of a SD difference in the non‐consistency indicator. Both low and high users of the internet are more likely to respond in ways that are not predictable to items in the same set of Likert‐type scales.

**Table 2:** The association between internet use and indicators of questionnaire response behaviour.

Variable	Non Response	Non Differentiation	Non Consistency
Constant	−0.11	1.26	−0.55
	(0.42)	(0.65)	(0.68)
Girl	−0.02*	‐0.01	−0.12***
	(0.01)	(0.03)	(0.03)
Age	‐0.02	−0.07	0.02
	(0.03)	(0.04)	(0.04)
ESCS Index	−0.03**	−0.01	−0.06***
	(0.01)	(0.02)	(0.02)
Low Internet User (vs. Medium)	0.11	0.16	0.32***
	(0.06)	(0.09)	(0.08)
High Internet User (vs. Medium)	−0.01	−0.02	0.12***
	(0.02)	(0.03)	(0.02)
Reading Fluency	0.00***	0.00	0.00**
	(0.00)	(0.00)	(0.00)
*N* (students)	151,092	151,092	151,092

*Note:* Results report OLS regression coefficients of regressions estimating each of the three questionnaire‐based perseverance indicators separately as a function of students’ background characteristics. ESCS Index stands for the PISA Index of Economic, Social and Cultural Status. The table presents average results across equally‐weighted country results. Standard errors in parenthesis.

** *p* < 5%;

*** *p* < 1%.

## 5 Conclusions

Education systems are required to ensure that young people develop new literacies as well as the capacity to use them successfully to solve problems whether on‐ or off‐line (Leu et al. 2014). As technology progresses, individuals will be increasingly expected to solve problems on digital platforms and will be rewarded for being able to do so in the labor market and beyond (OECD 2015). At the same time, educators, politicians, and parents express concern over the possible consequences of internet use (Orben 2020) and worry that a high use of connected devices reduces students' skills development and task persistence (Lissak 2018). Whereas many countries have invested heavily in digital technologies in education (UNESCO 2023), some are now reducing the use of connected applications in schools (Swedish Ministry of Education and Research 2023; Ministere de l'Education Nationale 2024).

Our findings partly align with earlier results indicating that high levels of internet use are associated with lower levels of achievement in reading (Vedechkina and Borgonovi 2021; Borgonovi and Pokropek 2021). In fact, we found that students who use the internet heavily display lower baseline reading accuracy compared to moderate users. Furthermore, our findings reinforce the evidence base on the “inverted U” hypothesis for reading achievement, whereby extremely low or extremely high levels of internet use are associated with somewhat lower accuracy compared to moderate use (Coiro 2011; Borgonovi and Pokropek 2021).

However, our results add an important nuance to the literature. Using PISA's item rotation design, we offer new insights on how adolescents' accuracy evolves during a protracted digital low‐stake assessment. More specifically we show that young people who are heavy internet users do not experience a stronger decline in accuracy over the course of the test. In fact, our analyses point to a marginally smaller decline for heavy internet users—i.e., they are less likely to suffer large drops in accuracy between the start and the end of the assessment. These results stand in contrast to some studies warning that extensive engagement with digital media might lead to a reduced ability to sustain attention (Alimoradi et al. 2019) and suggest, instead, that for heavy users the potential drawbacks in initial achievement might be partially offset by motivational or familiarity factors while performing digital academic tasks which may influence their ability to better sustain baseline levels of accuracy over the course of the assessment (Ettinger and Cohen 2020).

We also illustrate that students with different levels of internet use are equally likely to skip multiple‐choice questions in the PISA background questionnaire and to provide undifferentiated responses. By contrast, low and high users of the internet are more likely than students with medium levels of internet use to respond in ways that are not predictable to items in the same set of Likert‐type scales. These results suggest that findings identified in the main set of analyses are unlikely to reflect systematic underlying differences in measures of engagement that have been shown in the literature to be strongly associated with personality traits like conscientiousness (Brunello et al. 2021) but, rather, their capacity to continue engaging purposefully in cognitively demanding digital tasks.

Our interpretation of findings on changes in accuracy as a function of item position as evidence on the association between internet use and task persistence relies on one plausible mechanism—that reduced declines in accuracy toward the end of the test reflect sustained engagement. Other explanations cannot be ruled out. Although we control for item difficulty, the adaptive design of PISA for reading may mean that students' fatigue over the course of the assessment could vary given the range of items with different level of difficulty they were exposed to. We cannot completely rule out this possibility. Nonetheless, we partly addressed this concern by comparing the decline in accuracy of students who were administered, by chance, either the harder or the easier item set after they had similar levels of accuracy in the first stage and results are aligned with those presented.

Previous research suggests that large declines in accuracy toward the end of an assessment are associated with poorer attention and lower executive functioning (Levine et al. 2007) and worse long‐term outcomes independently of baseline levels of accuracy (Borgonovi et al. 2021). We find that students who rarely use the internet in fact show the steepest drops in accuracy over time, whereas those with high levels of use exhibit the lowest drop. This pattern holds even after controlling for student background, reading fluency, and time spent per item. These results broaden prior evidence by indicating that, in certain contexts, high internet use might be linked to digital‐task stamina, although it remains possible that non‐academically oriented digital habits undermine reading accuracy and the ability to sustain accuracy in other ways (Prensky 2001; Kirschner and De Bruyckere 2017).

A key strength of our work is that we use data from an internationally validated, low‐stake digital assessment administered in classroom settings to large, representative samples of students who completed the test under the same conditions. Tasks administered in PISA reflect the set of tasks 15‐year‐old students can be expected to be asked to solve in education settings and reflect the set of tasks they may encounter to make informed decisions in their lives (OECD 2019a). Furthermore, the measures of accuracy and the analytical strategy to identify drop in accuracy that we used in our study have been previously validated for cross‐country comparisons and have been shown to independently predict young people's educational progress (Borgonovi et al. 2021).

The literature indicates that individuals who are capable of completing demanding tasks are characterised by both distinct personality traits, such as persistence, as well as more task‐specific motivational drivers, such as self‐efficacy and valence (Schunk and DiBenedetto 2020). We show that students with different levels of internet use do not differ markedly from other students with respect to their adoption of other behaviours that are indicative of underlying personality traits such as conscientiousness.

Another important point relates to the relatively small group of low internet users (6% of the sample). Although they form a minority of students in most countries, they displayed both lower baseline accuracy and greater declines in accuracy relative to other groups. This group's small size suggests that educators might overlook them. However, our findings underscore the need for targeted support. For instance, these students may lack familiarity with digital tools and applications or broader study habits that would help them maintain attention over lengthy tasks. Their relatively small size underscores that any interventions aimed specifically at low‐use students would be more narrowly focused, but potentially highly beneficial, given that they appear the most vulnerable to performance drops near the end of assessments. Future research might explore the underlying causes for low internet users' steeper declines, such as limited access to technology or different motivational profiles, to design more effective educational strategies that mitigate performance gaps.

Our study is not causal and therefore cannot speak about the relative merit of limiting/promoting internet use in schools to aid achievement and promote task persistence. At the same time, when combining our results obtained with large, representative samples with experimental evidence on behavioural and psychological characteristics that determine accuracy in cognitive tasks and ability to sustain baseline levels of accuracy while completing long cognitive tasks, it is possible to suggest that moderate levels of internet use do not prevent the acquisition of literacy among adolescents and do not hinder the ability to sustain accuracy over time.

### 5.1 Limitations and Future Research

Our study suffers from a number of limitations that future research should address to strengthen the evidence base on the relationship between internet use, accuracy and ability to sustain accuracy in long cognitive tasks. The first set of limitations concerns the focus of our analyses. Although we used data from a large number of countries, these countries are rather homogeneous in terms of economic development and baseline accuracy. Associations may differ in other contexts, and as the PISA country coverage expands, it will become possible to examine these associations in a wider range of environments. In addition, while prior research and our findings highlight differences between boys and girls in accuracy, perseverance, and levels of internet use, exploring gender‐based heterogeneity in the association between internet use, accuracy, and perseverance was beyond the scope of this study. Future research could investigate these differences in greater detail, as well as how they manifest across different academic domains.

The second set of limitations is methodological. Because students self‐reported their levels of internet use, our measure may be subject to bias, potentially linked to their baseline accuracy or ability to sustain baseline levels of accuracy over time. Future studies could employ observational measures of internet use, such as having participants activate screen‐time features on their phone or other connected devices, to obtain objective usage data. A difficulty with this approach lies in combining more accurate measurement of internet use with the accurate large‐scale standardised measurement of academic achievement adopted in our study. Additional work is also needed to establish the causal nature of the relationships identified and to pinpoint the primary cognitive and motivational factors that influence the ability to sustain accuracy during long cognitive tasks. Finally, future research should explore why associations vary across countries and academic domains, shedding light on the context‐specific factors that drive these differences.

## Ethics Statement

Ethics approval from the University College London review board was sought and obtained for the project.

## Supporting information

Table S1 Country specific descriptive statistics. Figure S1. *Odds ratios associated with item position among students with low, medium and high use of the internet*. Notes: Countries are ranked in descending order of the Odds Ratios (ORs) of students responding correctly associated to a reading question when the question is placed at the start of the PISA test (first percentile) rather than the end (last percentile). Bars represent the ORs for students who are in the medium use of the internet group, and dots represent ORs for students who are in the high levels of internet use group and triangles represent ORs for students in the low levels of internet use group. The numbers in parenthesis next to the country code refer, respectively, to the % of students who are in the high use of the internet group and the % of students who are in the low use of the internet group. Dark dots and triangles indicate countries for which estimates of the interaction between level of internet use and item position is statistically significant at least at the 5% level (see main text for more details). Results are based on country‐specific results of Model 2a presented in Table 2 and available in the Supplementary Online Annex 2.

**Supplementary Online Annex 2 Country specific Results**.

## Data Availability

The data that support the findings of this study are available in PISA 2018 Database at https://www.oecd.org/en/data/datasets/pisa-2018-database.html.

## Endnotes

1 As an additional robustness check, we estimated our key model on the subsample of students who were randomly assigned to lower or higher difficulty items in the second part of the assessment (medium‐achieving students). Comparing estimates of declines in accuracy for these students allows to account for the potential cumulative effect on accuracy of being exposed to a series of more difficult or easier items. We did not find that such cumulative effect played a role. Results can be requested from the author.
