# Supplementary material for: The Relationship Between Internet Use, Achievement, and Persistence in Digital Tasks
Source: J Adolesc. 2025 Apr 13;97(5):1373–84. doi: 10.1002/jad.12503 (PMC12217420; doi:10.1002/jad.12503)
Supplement: Supplementary file 1 — Table S1 Country specific descriptive statistics. Figure S1. Odds ratios associated with item position among students with low, medium and high use of the internet. Notes: Countries are ranked in descending order of the Odds Ratios (ORs) of students responding correctly associated to a reading question when the question is placed at the start of the PISA test (first percentile) rather than the end (last percentile). Bars represent the ORs for students who are in the medium use of the internet group, and dots represent ORs for students who are in the high levels of internet use group and triangles represent ORs for students in the low levels of internet use group. The numbers in parenthesis next to the country code refer, respectively, to the % of students who are in the high use of the internet group and the % of students who are in the low use of the internet group. Dark dots and triangles indicate countries for which estimates of the interaction between level of internet use and item position is statistically significant at least at the 5% level (see main text for more details). Results are based on country‐specific results of Model 2a presented in Table 2 and available in the Supplementary Online Annex 2. [file JAD-97-1373-s002.docx]

**Supplementary Online Annex 1**

Table S1 Country specific descriptive statistics

| Country | N | Sample  Selection | Mis-routed (%) | Female Share | Age | ESCS | Reading fluency (seconds) | Low internet user (%) | Medium internet user (%) | High internet user (%) |
| --- | --- | --- | --- | --- | --- | --- | --- | --- | --- | --- |
| AUS | 7388 | 62.94 | 8.51 | 0.50 | 15.78 | 0.32 | 85449 | 3.49 | 52.84 | 43.67 |
| AUT | 5368 | 92.71 | 8.61 | 0.50 | 15.81 | 0.09 | 87610 | 4.71 | 56.67 | 38.62 |
| BEL | 5382 | 79.25 | 8.10 | 0.52 | 15.85 | 0.25 | 90113 | 3.12 | 53.21 | 43.66 |
| CZE | 5630 | 94.11 | 8.15 | 0.50 | 15.81 | -0.08 | 89866 | 5.13 | 62.06 | 32.81 |
| DNK | 4719 | 75.36 | 7.78 | 0.50 | 15.76 | 0.58 | 93029 | 1.46 | 52.47 | 46.07 |
| EST | 3967 | 85.95 | 8.48 | 0.51 | 15.83 | 0.09 | 85811 | 2.60 | 58.36 | 39.05 |
| FIN | 4291 | 90.56 | 9.40 | 0.50 | 15.72 | 0.33 | 82215 | 1.91 | 59.89 | 38.20 |
| FRA | 4264 | 81.40 | 8.88 | 0.50 | 15.87 | 0.04 | 99158 | 5.91 | 55.30 | 38.79 |
| GBR | 5568 | 80.43 | 8.48 | 0.51 | 15.76 | 0.28 | 82737 | 1.76 | 46.44 | 51.80 |
| GRC | 4640 | 85.88 | 7.58 | 0.51 | 15.7 | 0.01 | 91489 | 8.15 | 56.36 | 35.50 |
| HUN | 4384 | 95.90 | 7.65 | 0.51 | 15.78 | -0.07 | 88668 | 4.33 | 54.22 | 41.45 |
| IRL | 3755 | 75.80 | 8.69 | 0.49 | 15.71 | 0.13 | 85981 | 3.36 | 55.79 | 40.85 |
| ISL | 2335 | 86.02 | 8.11 | 0.51 | 15.76 | 0.59 | 97314 | 1.67 | 59.53 | 38.80 |
| ISR | 3544 | 77.00 | 8.97 | 0.54 | 15.7 | 0.36 | 85780 | 8.58 | 51.44 | 39.98 |
| ITA | 8570 | 85.85 | 7.29 | 0.49 | 15.78 | -0.15 | 91138 | 5.68 | 50.91 | 43.41 |
| JPN | 5289 | 98.01 | 8.74 | 0.51 | 15.78 | -0.1 | 95546 | 13.10 | 69.48 | 17.41 |
| KOR | 5837 | 98.19 | 9.01 | 0.48 | 15.73 | 0.08 | 95104 | 16.89 | 66.59 | 16.52 |
| LTU | 5581 | 94.04 | 7.46 | 0.50 | 15.81 | 0.03 | 96268 | 5.45 | 47.79 | 46.77 |
| LVA | 4087 | 92.02 | 7.63 | 0.51 | 15.79 | -0.03 | 98188 | 4.26 | 52.02 | 43.72 |
| MEX | 5173 | 84.67 | 7.05 | 0.53 | 15.85 | -0.99 | 106676 | 17.84 | 42.30 | 39.86 |
| NZL | 3579 | 65.83 | 8.54 | 0.53 | 15.78 | 0.15 | 85233 | 3.44 | 50.55 | 46.02 |
| POL | 4749 | 97.25 | 8.55 | 0.52 | 15.73 | -0.14 | 86021 | 3.75 | 53.09 | 43.17 |
| SVK | 4769 | 95.49 | 7.62 | 0.51 | 15.81 | -0.12 | 87663 | 7.21 | 52.30 | 40.49 |
| SVN | 4842 | 89.31 | 8.66 | 0.48 | 15.73 | 0.05 | 87686 | 6.36 | 63.69 | 29.95 |
| SWE | 3374 | 75.73 | 9.28 | 0.51 | 15.72 | 0.49 | 94278 | 0.83 | 43.33 | 55.84 |
| TUR | 5721 | 95.90 | 6.80 | 0.49 | 15.83 | -1.14 | 82810 | 16.66 | 53.84 | 29.51 |
| USA | 3109 | 75.50 | 8.36 | 0.50 | 15.84 | 0.18 | 82700 | 4.18 | 46.29 | 49.53 |

Figure S1

*Odds ratios associated with item position among students with low, medium and high use of the internet*


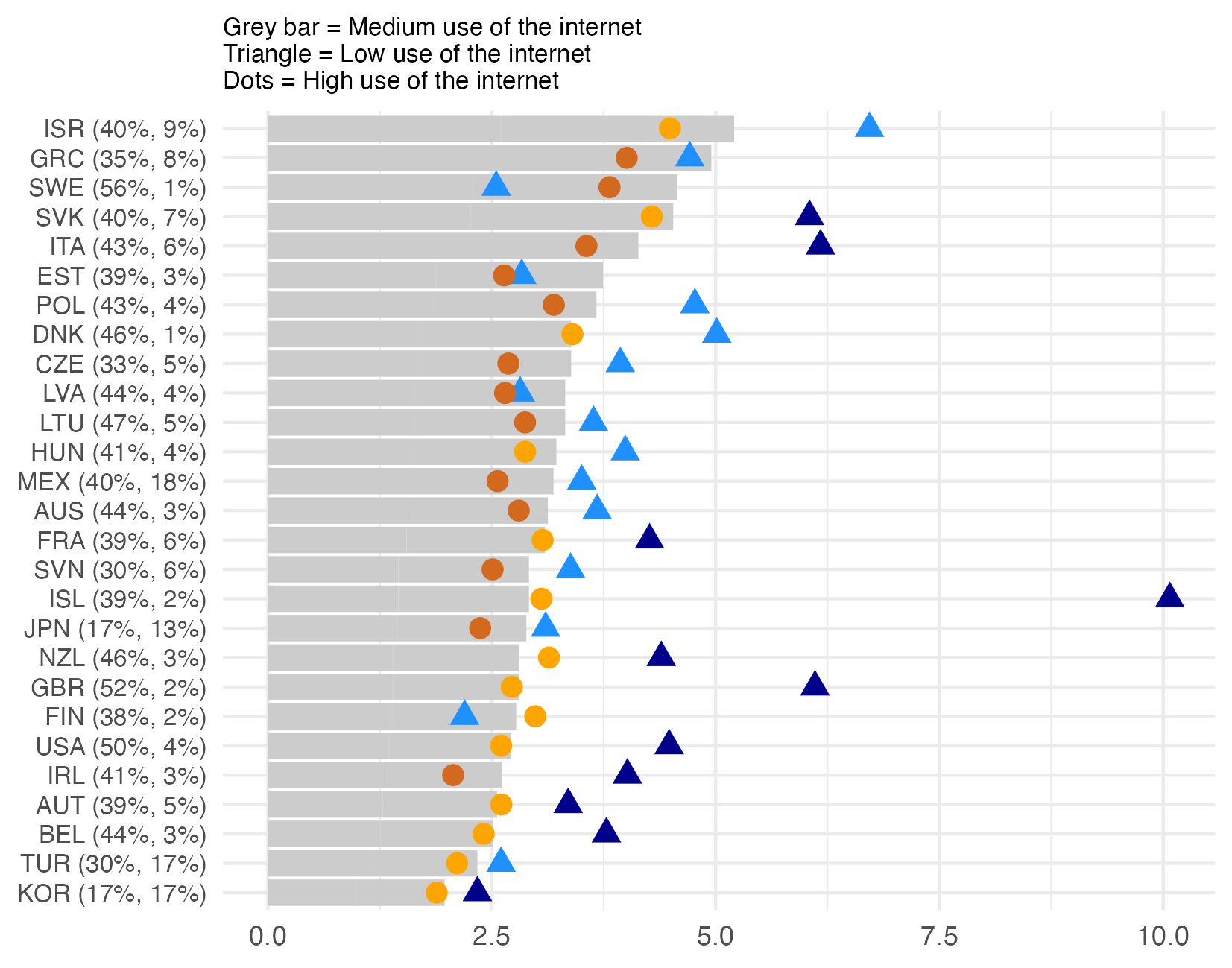


OR

Notes: Countries are ranked in descending order of the Odds Ratios (ORs) of students responding correctly associated to a reading question when the question is placed at the start of the PISA test (first percentile) rather than the end (last percentile). Bars represent the ORs for students who are in the medium use of the internet group, and dots represent ORs for students who are in the high levels of internet use group and triangles represent ORs for students in the low levels of internet use group. The numbers in parenthesis next to the country code refer, respectively, to the % of students who are in the high use of the internet group and the % of students who are in the low use of the internet group. Dark dots and triangles indicate countries for which estimates of the interaction between level of internet use and item position is statistically significant at least at the 5% level (see main text for more details). Results are based on country-specific results of Model 2a presented in Table 2 and available in the Supplementary Online Annex 2.
