# Supplementary material for: The Relationship Between Internet Use, Achievement, and Persistence in Digital Tasks
Source: J Adolesc. 2025 Apr 13;97(5):1373–84. doi: 10.1002/jad.12503 (PMC12217420; doi:10.1002/jad.12503)
Supplement: Supplementary file 2 — Supplementary Online Annex 2 Country specific Results. [file JAD-97-1373-s001.docx]

**Supplementary Online Annex 2 Country specific Results**

|  | AUS | AUS | AUS | AUS | AUS | AUT | AUT | AUT | AUT | AUT |
| --- | --- | --- | --- | --- | --- | --- | --- | --- | --- | --- |
| Constant | 2.656^***^ | 2.677^***^ | 0.644^***^ | 1.129^***^ | 1.155^***^ | 2.743^***^ | 2.733^***^ | 0.716^***^ | 1.066^***^ | 1.056^***^ |
|  | (0.106) | (0.107) | (0.115) | (0.144) | (0.145) | (0.126) | (0.127) | (0.137) | (0.169) | (0.169) |
| female | 0.232^***^ | 0.232^***^ | 0.197^***^ | 0.199^***^ | 0.199^***^ | 0.158^***^ | 0.158^***^ | 0.123^***^ | 0.124^***^ | 0.124^***^ |
|  | (0.029) | (0.029) | (0.028) | (0.028) | (0.028) | (0.033) | (0.033) | (0.031) | (0.031) | (0.031) |
| age | 0.217^***^ | 0.217^***^ | 0.217^***^ | 0.217^***^ | 0.217^***^ | 0.403^***^ | 0.403^***^ | 0.385^***^ | 0.385^***^ | 0.385^***^ |
|  | (0.050) | (0.050) | (0.047) | (0.047) | (0.047) | (0.051) | (0.051) | (0.048) | (0.048) | (0.048) |
| escs_scale | 0.369^***^ | 0.369^***^ | 0.360^***^ | 0.360^***^ | 0.360^***^ | 0.142^***^ | 0.142^***^ | 0.144^***^ | 0.143^***^ | 0.143^***^ |
|  | (0.017) | (0.017) | (0.016) | (0.016) | (0.016) | (0.019) | (0.019) | (0.018) | (0.018) | (0.018) |
| reading_fluency | -0.008^***^ | -0.008^***^ | -0.008^***^ | -0.008^***^ | -0.008^***^ | -0.008^***^ | -0.008^***^ | -0.009^***^ | -0.009^***^ | -0.009^***^ |
|  | (0.001) | (0.001) | (0.0005) | (0.0005) | (0.0005) | (0.001) | (0.001) | (0.0005) | (0.0005) | (0.0005) |
| ICT_excess_use_cat1high | -0.245^***^ | -0.297^***^ | -0.228^***^ | -0.228^***^ | -0.288^***^ | -0.216^***^ | -0.208^***^ | -0.192^***^ | -0.192^***^ | -0.185^***^ |
|  | (0.030) | (0.042) | (0.028) | (0.028) | (0.041) | (0.030) | (0.046) | (0.028) | (0.028) | (0.045) |
| ICT_excess_use_cat1low | -0.396^***^ | -0.315^***^ | -0.365^***^ | -0.365^***^ | -0.288^**^ | -0.247^***^ | -0.111 | -0.229^***^ | -0.231^***^ | -0.096 |
|  | (0.080) | (0.117) | (0.075) | (0.075) | (0.114) | (0.067) | (0.106) | (0.063) | (0.063) | (0.103) |
| percentile_rank_round | -1.103^***^ | -1.147^***^ | -0.911^***^ | -1.792^***^ | -1.844^***^ | -0.967^***^ | -0.946^***^ | -0.763^***^ | -1.398^***^ | -1.377^***^ |
|  | (0.047) | (0.056) | (0.046) | (0.172) | (0.174) | (0.049) | (0.058) | (0.047) | (0.196) | (0.199) |
| timing_decile |  |  | 0.344^***^ | 0.258^***^ | 0.258^***^ |  |  | 0.346^***^ | 0.283^***^ | 0.283^***^ |
|  |  |  | (0.008) | (0.018) | (0.018) |  |  | (0.010) | (0.020) | (0.020) |
| timing_squ_scale |  |  | -0.025^***^ | -0.020^***^ | -0.020^***^ |  |  | -0.024^***^ | -0.021^***^ | -0.021^***^ |
|  |  |  | (0.001) | (0.002) | (0.002) |  |  | (0.001) | (0.002) | (0.002) |
| ICT_excess_use_cat1high:percentile_rank_round |  | 0.110^*^ |  |  | 0.125^**^ |  | -0.018 |  |  | -0.014 |
|  |  | (0.064) |  |  | (0.062) |  | (0.073) |  |  | (0.070) |
| ICT_excess_use_cat1low:percentile_rank_round |  | -0.162 |  |  | -0.147 |  | -0.278^*^ |  |  | -0.270^*^ |
|  |  | (0.175) |  |  | (0.169) |  | (0.168) |  |  | (0.162) |
| percentile_rank_round:timing_decile |  |  |  | 0.159^***^ | 0.159^***^ |  |  |  | 0.115^***^ | 0.115^***^ |
|  |  |  |  | (0.030) | (0.030) |  |  |  | (0.035) | (0.035) |
| percentile_rank_round:timing_squ_scale |  |  |  | -0.009^***^ | -0.009^***^ |  |  |  | -0.005^*^ | -0.005^*^ |
|  |  |  |  | (0.003) | (0.003) |  |  |  | (0.003) | (0.003) |
|  |  | | | | |  | | | | |
| question FE | Yes | Yes | Yes | Yes | Yes | Yes | Yes | Yes | Yes | Yes |
|  |  | | | | |  | | | | |
| Observations | 261.585 | 261.585 | 259.365 | 259.365 | 259.365 | 187.101 | 187.101 | 185.499 | 185.499 | 185.499 |
| Log Likelihood | -132,828.400 | -132,826.100 | -130,631.000 | -130,584.100 | -130,581.200 | -96,523.650 | -96,522.250 | -94,847.760 | -94,820.080 | -94,818.660 |
| Akaike Inf. Crit. | 266,162.800 | 266,162.100 | 261,772.000 | 261,682.200 | 261,680.400 | 193,553.300 | 193,554.500 | 190,205.500 | 190,154.200 | 190,155.300 |
| Bayesian Inf. Crit. | 268,812.800 | 268,833.100 | 264,440.900 | 264,372.000 | 264,391.100 | 196,118.600 | 196,140.000 | 192,788.900 | 192,757.800 | 192,779.200 |

|  | BEL | BEL | BEL | BEL | BEL | CZE | CZE | CZE | CZE | CZE |
| --- | --- | --- | --- | --- | --- | --- | --- | --- | --- | --- |
| Constant | 2.414^***^ | 2.417^***^ | 1.016^***^ | 1.424^***^ | 1.432^***^ | 2.699^***^ | 2.734^***^ | 0.955^***^ | 1.595^***^ | 1.629^***^ |
|  | (0.122) | (0.122) | (0.132) | (0.164) | (0.165) | (0.128) | (0.129) | (0.139) | (0.170) | (0.170) |
| female | 0.041 | 0.041 | 0.030 | 0.031 | 0.031 | 0.129^***^ | 0.128^***^ | 0.111^***^ | 0.111^***^ | 0.110^***^ |
|  | (0.028) | (0.028) | (0.027) | (0.027) | (0.027) | (0.028) | (0.028) | (0.027) | (0.027) | (0.027) |
| age | 0.117^***^ | 0.119^***^ | 0.113^**^ | 0.113^***^ | 0.115^***^ | -0.059 | -0.056 | -0.058 | -0.055 | -0.052 |
|  | (0.045) | (0.045) | (0.044) | (0.044) | (0.044) | (0.055) | (0.055) | (0.053) | (0.053) | (0.053) |
| escs_scale | 0.237^***^ | 0.237^***^ | 0.233^***^ | 0.233^***^ | 0.233^***^ | 0.219^***^ | 0.220^***^ | 0.212^***^ | 0.211^***^ | 0.212^***^ |
|  | (0.018) | (0.018) | (0.017) | (0.017) | (0.017) | (0.018) | (0.018) | (0.017) | (0.017) | (0.017) |
| reading_fluency | -0.008^***^ | -0.008^***^ | -0.008^***^ | -0.008^***^ | -0.008^***^ | -0.007^***^ | -0.007^***^ | -0.007^***^ | -0.007^***^ | -0.007^***^ |
|  | (0.0005) | (0.0005) | (0.0005) | (0.0005) | (0.0005) | (0.0005) | (0.0005) | (0.0005) | (0.0005) | (0.0005) |
| ICT_excess_use_cat1high | -0.235^***^ | -0.255^***^ | -0.228^***^ | -0.228^***^ | -0.261^***^ | -0.113^***^ | -0.222^***^ | -0.105^***^ | -0.105^***^ | -0.218^***^ |
|  | (0.028) | (0.043) | (0.027) | (0.027) | (0.043) | (0.029) | (0.045) | (0.028) | (0.028) | (0.044) |
| ICT_excess_use_cat1low | -0.416^***^ | -0.228^*^ | -0.416^***^ | -0.414^***^ | -0.236^**^ | -0.301^***^ | -0.231^**^ | -0.289^***^ | -0.288^***^ | -0.230^**^ |
|  | (0.076) | (0.120) | (0.074) | (0.074) | (0.119) | (0.059) | (0.095) | (0.057) | (0.057) | (0.094) |
| percentile_rank_round | -0.921^***^ | -0.927^***^ | -0.834^***^ | -1.602^***^ | -1.619^***^ | -1.156^***^ | -1.229^***^ | -0.986^***^ | -2.165^***^ | -2.236^***^ |
|  | (0.046) | (0.057) | (0.046) | (0.195) | (0.198) | (0.053) | (0.060) | (0.051) | (0.196) | (0.198) |
| timing_decile |  |  | 0.244^***^ | 0.170^***^ | 0.170^***^ |  |  | 0.300^***^ | 0.185^***^ | 0.185^***^ |
|  |  |  | (0.010) | (0.020) | (0.020) |  |  | (0.010) | (0.020) | (0.020) |
| timing_squ_scale |  |  | -0.018^***^ | -0.015^***^ | -0.015^***^ |  |  | -0.021^***^ | -0.015^***^ | -0.015^***^ |
|  |  |  | (0.001) | (0.002) | (0.002) |  |  | (0.001) | (0.002) | (0.002) |
| ICT_excess_use_cat1high:percentile_rank_round |  | 0.041 |  |  | 0.068 |  | 0.232^***^ |  |  | 0.235^***^ |
|  |  | (0.070) |  |  | (0.068) |  | (0.075) |  |  | (0.072) |
| ICT_excess_use_cat1low:percentile_rank_round |  | -0.411^**^ |  |  | -0.381^*^ |  | -0.150 |  |  | -0.121 |
|  |  | (0.201) |  |  | (0.197) |  | (0.160) |  |  | (0.154) |
| percentile_rank_round:timing_decile |  |  |  | 0.139^***^ | 0.139^***^ |  |  |  | 0.214^***^ | 0.213^***^ |
|  |  |  |  | (0.035) | (0.035) |  |  |  | (0.034) | (0.034) |
| percentile_rank_round:timing_squ_scale |  |  |  | -0.007^**^ | -0.007^**^ |  |  |  | -0.011^***^ | -0.011^***^ |
|  |  |  |  | (0.003) | (0.003) |  |  |  | (0.003) | (0.003) |
|  |  | | | | |  | | | | |
| question FE | Yes | Yes | Yes | Yes | Yes | Yes | Yes | Yes | Yes | Yes |
|  |  | | | | |  | | | | |
| Observations | 184.872 | 184.872 | 183.345 | 183.345 | 183.345 | 197.043 | 197.043 | 195.437 | 195.437 | 195.437 |
| Log Likelihood | -94,258.450 | -94,255.780 | -93,124.340 | -93,087.530 | -93,084.650 | -96,835.910 | -96,829.790 | -95,309.700 | -95,236.040 | -95,229.700 |
| Akaike Inf. Crit. | 189,022.900 | 189,021.600 | 186,758.700 | 186,689.100 | 186,687.300 | 194,175.800 | 194,167.600 | 191,127.400 | 190,984.100 | 190,975.400 |
| Bayesian Inf. Crit. | 191,585.100 | 191,604.100 | 189,339.100 | 189,289.700 | 189,308.200 | 196,744.000 | 196,756.100 | 193,713.900 | 193,590.900 | 193,602.600 |
|  |  |  |  |  |  |  |  |  |  |  |

|  | DNK | DNK | DNK | DNK | DNK | EST | EST | EST | EST | EST |
| --- | --- | --- | --- | --- | --- | --- | --- | --- | --- | --- |
| Constant | 2.749^***^ | 2.745^***^ | 0.835^***^ | 1.438^***^ | 1.443^***^ | 3.559^***^ | 3.635^***^ | 2.147^***^ | 2.286^***^ | 2.368^***^ |
|  | (0.125) | (0.126) | (0.137) | (0.173) | (0.174) | (0.169) | (0.170) | (0.179) | (0.214) | (0.215) |
| female | 0.218^***^ | 0.218^***^ | 0.193^***^ | 0.194^***^ | 0.194^***^ | 0.299^***^ | 0.297^***^ | 0.278^***^ | 0.279^***^ | 0.277^***^ |
|  | (0.031) | (0.031) | (0.030) | (0.030) | (0.030) | (0.034) | (0.034) | (0.033) | (0.033) | (0.033) |
| age | 0.127^**^ | 0.127^**^ | 0.139^***^ | 0.139^***^ | 0.139^***^ | 0.189^***^ | 0.187^***^ | 0.189^***^ | 0.188^***^ | 0.186^***^ |
|  | (0.055) | (0.055) | (0.053) | (0.053) | (0.053) | (0.059) | (0.059) | (0.057) | (0.057) | (0.057) |
| escs_scale | 0.391^***^ | 0.391^***^ | 0.381^***^ | 0.380^***^ | 0.380^***^ | 0.288^***^ | 0.288^***^ | 0.284^***^ | 0.284^***^ | 0.284^***^ |
|  | (0.024) | (0.024) | (0.022) | (0.022) | (0.022) | (0.023) | (0.023) | (0.022) | (0.022) | (0.022) |
| reading_fluency | -0.010^***^ | -0.010^***^ | -0.010^***^ | -0.010^***^ | -0.010^***^ | -0.008^***^ | -0.008^***^ | -0.008^***^ | -0.008^***^ | -0.008^***^ |
|  | (0.001) | (0.001) | (0.001) | (0.001) | (0.001) | (0.001) | (0.001) | (0.001) | (0.001) | (0.001) |
| ICT_excess_use_cat1high | -0.189^***^ | -0.188^***^ | -0.176^***^ | -0.176^***^ | -0.192^***^ | -0.185^***^ | -0.364^***^ | -0.178^***^ | -0.177^***^ | -0.362^***^ |
|  | (0.032) | (0.048) | (0.031) | (0.031) | (0.047) | (0.035) | (0.056) | (0.034) | (0.034) | (0.055) |
| ICT_excess_use_cat1low | -0.278^**^ | -0.102 | -0.253^**^ | -0.254^**^ | -0.141 | -0.366^***^ | -0.507^***^ | -0.342^***^ | -0.339^***^ | -0.496^***^ |
|  | (0.132) | (0.197) | (0.126) | (0.126) | (0.193) | (0.106) | (0.167) | (0.104) | (0.104) | (0.167) |
| percentile_rank_round | -1.230^***^ | -1.223^***^ | -1.067^***^ | -2.172^***^ | -2.183^***^ | -1.178^***^ | -1.327^***^ | -1.096^***^ | -1.308^***^ | -1.464^***^ |
|  | (0.058) | (0.068) | (0.055) | (0.210) | (0.214) | (0.058) | (0.068) | (0.058) | (0.229) | (0.232) |
| timing_decile |  |  | 0.332^***^ | 0.223^***^ | 0.224^***^ |  |  | 0.248^***^ | 0.223^***^ | 0.223^***^ |
|  |  |  | (0.010) | (0.022) | (0.022) |  |  | (0.011) | (0.024) | (0.024) |
| timing_squ_scale |  |  | -0.025^***^ | -0.019^***^ | -0.019^***^ |  |  | -0.019^***^ | -0.020^***^ | -0.020^***^ |
|  |  |  | (0.001) | (0.002) | (0.002) |  |  | (0.001) | (0.002) | (0.002) |
| ICT_excess_use_cat1high:percentile_rank_round |  | -0.004 |  |  | 0.034 |  | 0.350^***^ |  |  | 0.352^***^ |
|  |  | (0.077) |  |  | (0.075) |  | (0.084) |  |  | (0.083) |
| ICT_excess_use_cat1low:percentile_rank_round |  | -0.392 |  |  | -0.242 |  | 0.278 |  |  | 0.305 |
|  |  | (0.325) |  |  | (0.314) |  | (0.255) |  |  | (0.252) |
| percentile_rank_round:timing_decile |  |  |  | 0.201^***^ | 0.200^***^ |  |  |  | 0.039 | 0.040 |
|  |  |  |  | (0.037) | (0.037) |  |  |  | (0.040) | (0.040) |
| percentile_rank_round:timing_squ_scale |  |  |  | -0.010^***^ | -0.010^***^ |  |  |  | 0.002 | 0.002 |
|  |  |  |  | (0.003) | (0.003) |  |  |  | (0.004) | (0.004) |
|  |  | | | | |  | | | | |
| question FE | Yes | Yes | Yes | Yes | Yes | Yes | Yes | Yes | Yes | Yes |
|  |  | | | | |  | | | | |
| Observations | 164.407 | 164.407 | 162.99 | 162.99 | 162.99 | 139.175 | 139.175 | 138.011 | 138.011 | 138.011 |
| Log Likelihood | -84,829.130 | -84,828.380 | -83,438.080 | -83,379.880 | -83,379.420 | -68,934.880 | -68,925.680 | -68,137.010 | -68,113.650 | -68,103.970 |
| Akaike Inf. Crit. | 170,160.300 | 170,162.800 | 167,382.200 | 167,269.800 | 167,272.800 | 138,375.800 | 138,361.400 | 136,784.000 | 136,741.300 | 136,725.900 |
| Bayesian Inf. Crit. | 172,672.800 | 172,695.300 | 169,912.500 | 169,820.100 | 169,843.200 | 140,866.200 | 140,871.400 | 139,292.000 | 139,268.900 | 139,273.200 |
|  |  |  |  |  |  |  |  |  |  |  |

|  | FIN | FIN | FIN | FIN | FIN | FRA | FRA | FRA | FRA | FRA |
| --- | --- | --- | --- | --- | --- | --- | --- | --- | --- | --- |
| Constant | 2.845^***^ | 2.833^***^ | 1.172^***^ | 1.569^***^ | 1.559^***^ | 2.576^***^ | 2.569^***^ | 0.799^***^ | 1.170^***^ | 1.161^***^ |
|  | (0.154) | (0.155) | (0.165) | (0.202) | (0.202) | (0.134) | (0.135) | (0.147) | (0.186) | (0.187) |
| female | 0.492^***^ | 0.492^***^ | 0.455^***^ | 0.456^***^ | 0.456^***^ | 0.101^***^ | 0.102^***^ | 0.082^***^ | 0.082^***^ | 0.083^***^ |
|  | (0.035) | (0.035) | (0.034) | (0.034) | (0.034) | (0.031) | (0.031) | (0.029) | (0.029) | (0.029) |
| age | 0.305^***^ | 0.305^***^ | 0.313^***^ | 0.312^***^ | 0.312^***^ | 0.146^***^ | 0.146^***^ | 0.136^***^ | 0.134^***^ | 0.133^***^ |
|  | (0.061) | (0.061) | (0.058) | (0.058) | (0.058) | (0.053) | (0.053) | (0.051) | (0.050) | (0.050) |
| escs_scale | 0.397^***^ | 0.397^***^ | 0.387^***^ | 0.386^***^ | 0.387^***^ | 0.275^***^ | 0.275^***^ | 0.272^***^ | 0.271^***^ | 0.270^***^ |
|  | (0.024) | (0.024) | (0.023) | (0.023) | (0.023) | (0.021) | (0.021) | (0.020) | (0.020) | (0.020) |
| reading_fluency | -0.007^***^ | -0.007^***^ | -0.007^***^ | -0.007^***^ | -0.007^***^ | -0.009^***^ | -0.009^***^ | -0.010^***^ | -0.010^***^ | -0.010^***^ |
|  | (0.001) | (0.001) | (0.001) | (0.001) | (0.001) | (0.001) | (0.001) | (0.001) | (0.001) | (0.001) |
| ICT_excess_use_cat1high | -0.304^***^ | -0.268^***^ | -0.288^***^ | -0.288^***^ | -0.259^***^ | -0.239^***^ | -0.244^***^ | -0.221^***^ | -0.221^***^ | -0.224^***^ |
|  | (0.036) | (0.056) | (0.035) | (0.035) | (0.055) | (0.032) | (0.051) | (0.031) | (0.031) | (0.050) |
| ICT_excess_use_cat1low | -0.223^*^ | -0.345^*^ | -0.207^*^ | -0.204^*^ | -0.337^*^ | -0.372^***^ | -0.218^**^ | -0.356^***^ | -0.356^***^ | -0.219^**^ |
|  | (0.128) | (0.204) | (0.123) | (0.123) | (0.202) | (0.067) | (0.107) | (0.064) | (0.064) | (0.104) |
| percentile_rank_round | -1.049^***^ | -1.025^***^ | -0.937^***^ | -1.654^***^ | -1.635^***^ | -1.149^***^ | -1.132^***^ | -0.967^***^ | -1.638^***^ | -1.618^***^ |
|  | (0.060) | (0.070) | (0.059) | (0.228) | (0.231) | (0.059) | (0.069) | (0.055) | (0.227) | (0.230) |
| timing_decile |  |  | 0.294^***^ | 0.223^***^ | 0.223^***^ |  |  | 0.304^***^ | 0.238^***^ | 0.238^***^ |
|  |  |  | (0.011) | (0.024) | (0.024) |  |  | (0.011) | (0.023) | (0.023) |
| timing_squ_scale |  |  | -0.022^***^ | -0.019^***^ | -0.019^***^ |  |  | -0.021^***^ | -0.018^***^ | -0.019^***^ |
|  |  |  | (0.001) | (0.002) | (0.002) |  |  | (0.001) | (0.002) | (0.002) |
| ICT_excess_use_cat1high:percentile_rank_round |  | -0.074 |  |  | -0.057 |  | 0.009 |  |  | 0.006 |
|  |  | (0.086) |  |  | (0.084) |  | (0.083) |  |  | (0.080) |
| ICT_excess_use_cat1low:percentile_rank_round |  | 0.233 |  |  | 0.245 |  | -0.322^*^ |  |  | -0.280^*^ |
|  |  | (0.304) |  |  | (0.298) |  | (0.175) |  |  | (0.168) |
| percentile_rank_round:timing_decile |  |  |  | 0.130^***^ | 0.130^***^ |  |  |  | 0.121^***^ | 0.120^***^ |
|  |  |  |  | (0.040) | (0.040) |  |  |  | (0.040) | (0.040) |
| percentile_rank_round:timing_squ_scale |  |  |  | -0.006^*^ | -0.006^*^ |  |  |  | -0.003 | -0.003 |
|  |  |  |  | (0.004) | (0.004) |  |  |  | (0.003) | (0.003) |
|  |  | | | | |  | | | | |
| question FE | Yes | Yes | Yes | Yes | Yes | Yes | Yes | Yes | Yes | Yes |
|  |  | | | | |  | | | | |
| Observations | 149.302 | 149.302 | 148.058 | 148.058 | 148.058 | 145.207 | 145.207 | 143.976 | 143.976 | 143.976 |
| Log Likelihood | -72,317.260 | -72,316.470 | -71,351.680 | -71,326.960 | -71,326.290 | -74,525.630 | -74,523.740 | -73,295.480 | -73,248.950 | -73,247.420 |
| Akaike Inf. Crit. | 145,140.500 | 145,142.900 | 143,213.400 | 143,167.900 | 143,170.600 | 149,557.300 | 149,557.500 | 147,101.000 | 147,011.900 | 147,012.800 |
| Bayesian Inf. Crit. | 147,648.700 | 147,670.900 | 145,739.200 | 145,713.600 | 145,736.100 | 152,058.400 | 152,078.400 | 149,619.700 | 149,550.400 | 149,571.100 |
|  |  |  |  |  |  |  |  |  |  |  |

|  | GBR | GBR | GBR | GBR | GBR | GRC | GRC | GRC | GRC | GRC |
| --- | --- | --- | --- | --- | --- | --- | --- | --- | --- | --- |
| Constant | 2.653^***^ | 2.653^***^ | 0.799^***^ | 1.138^***^ | 1.147^***^ | 2.144^***^ | 2.184^***^ | 0.048 | 0.928^***^ | 0.971^***^ |
|  | (0.115) | (0.116) | (0.126) | (0.160) | (0.161) | (0.120) | (0.122) | (0.134) | (0.172) | (0.173) |
| female | 0.064^**^ | 0.064^**^ | 0.042 | 0.044 | 0.044 | 0.310^***^ | 0.310^***^ | 0.272^***^ | 0.271^***^ | 0.271^***^ |
|  | (0.031) | (0.031) | (0.029) | (0.029) | (0.029) | (0.032) | (0.032) | (0.030) | (0.030) | (0.030) |
| age | 0.299^***^ | 0.299^***^ | 0.287^***^ | 0.287^***^ | 0.287^***^ | 0.233^***^ | 0.234^***^ | 0.225^***^ | 0.222^***^ | 0.223^***^ |
|  | (0.054) | (0.054) | (0.051) | (0.051) | (0.051) | (0.056) | (0.056) | (0.053) | (0.052) | (0.052) |
| escs_scale | 0.299^***^ | 0.298^***^ | 0.292^***^ | 0.292^***^ | 0.291^***^ | 0.204^***^ | 0.204^***^ | 0.208^***^ | 0.207^***^ | 0.207^***^ |
|  | (0.018) | (0.018) | (0.018) | (0.018) | (0.018) | (0.021) | (0.021) | (0.019) | (0.019) | (0.019) |
| reading_fluency | -0.011^***^ | -0.011^***^ | -0.011^***^ | -0.011^***^ | -0.011^***^ | -0.008^***^ | -0.008^***^ | -0.008^***^ | -0.008^***^ | -0.008^***^ |
|  | (0.001) | (0.001) | (0.001) | (0.001) | (0.001) | (0.001) | (0.001) | (0.001) | (0.001) | (0.001) |
| ICT_excess_use_cat1high | -0.323^***^ | -0.336^***^ | -0.304^***^ | -0.303^***^ | -0.336^***^ | -0.201^***^ | -0.301^***^ | -0.173^***^ | -0.172^***^ | -0.273^***^ |
|  | (0.031) | (0.046) | (0.030) | (0.030) | (0.045) | (0.034) | (0.054) | (0.032) | (0.032) | (0.052) |
| ICT_excess_use_cat1low | -0.642^***^ | -0.271 | -0.636^***^ | -0.636^***^ | -0.320^*^ | -0.372^***^ | -0.397^***^ | -0.356^***^ | -0.354^***^ | -0.402^***^ |
|  | (0.117) | (0.176) | (0.112) | (0.112) | (0.173) | (0.060) | (0.096) | (0.057) | (0.056) | (0.093) |
| percentile_rank_round | -1.036^***^ | -1.038^***^ | -0.897^***^ | -1.522^***^ | -1.544^***^ | -1.522^***^ | -1.604^***^ | -1.184^***^ | -2.860^***^ | -2.943^***^ |
|  | (0.052) | (0.065) | (0.050) | (0.198) | (0.202) | (0.062) | (0.073) | (0.059) | (0.220) | (0.223) |
| timing_decile |  |  | 0.317^***^ | 0.256^***^ | 0.256^***^ |  |  | 0.339^***^ | 0.182^***^ | 0.182^***^ |
|  |  |  | (0.010) | (0.020) | (0.020) |  |  | (0.011) | (0.022) | (0.022) |
| timing_squ_scale |  |  | -0.023^***^ | -0.021^***^ | -0.021^***^ |  |  | -0.021^***^ | -0.012^***^ | -0.012^***^ |
|  |  |  | (0.001) | (0.002) | (0.002) |  |  | (0.001) | (0.002) | (0.002) |
| ICT_excess_use_cat1high:percentile_rank_round |  | 0.028 |  |  | 0.069 |  | 0.212^**^ |  |  | 0.201^**^ |
|  |  | (0.073) |  |  | (0.070) |  | (0.089) |  |  | (0.083) |
| ICT_excess_use_cat1low:percentile_rank_round |  | -0.780^***^ |  |  | -0.644^**^ |  | 0.050 |  |  | 0.094 |
|  |  | (0.276) |  |  | (0.268) |  | (0.155) |  |  | (0.145) |
| percentile_rank_round:timing_decile |  |  |  | 0.113^***^ | 0.113^***^ |  |  |  | 0.302^***^ | 0.302^***^ |
|  |  |  |  | (0.035) | (0.035) |  |  |  | (0.038) | (0.038) |
| percentile_rank_round:timing_squ_scale |  |  |  | -0.004 | -0.004 |  |  |  | -0.017^***^ | -0.017^***^ |
|  |  |  |  | (0.003) | (0.003) |  |  |  | (0.003) | (0.003) |
|  |  | | | | |  | | | | |
| question FE | Yes | Yes | Yes | Yes | Yes | Yes | Yes | Yes | Yes | Yes |
|  |  | | | | |  | | | | |
| Observations | 193.202 | 193.202 | 191.587 | 191.587 | 191.587 | 158.994 | 158.994 | 157.514 | 157.514 | 157.514 |
| Log Likelihood | -99,149.160 | -99,144.590 | -97,596.020 | -97,552.590 | -97,548.570 | -84,406.250 | -84,403.240 | -82,566.540 | -82,462.820 | -82,459.710 |
| Akaike Inf. Crit. | 198,804.300 | 198,799.200 | 195,702.000 | 195,619.200 | 195,615.100 | 169,318.500 | 169,316.500 | 165,643.100 | 165,439.600 | 165,437.400 |
| Bayesian Inf. Crit. | 201,377.700 | 201,392.900 | 198,293.600 | 198,231.100 | 198,247.400 | 171,842.600 | 171,860.500 | 168,184.700 | 168,001.200 | 168,019.000 |
|  |  |  |  |  |  |  |  |  |  |  |

|  | HUN | HUN | HUN | HUN | HUN | IRL | IRL | IRL | IRL | IRL |
| --- | --- | --- | --- | --- | --- | --- | --- | --- | --- | --- |
| Constant | 2.030^***^ | 2.050^***^ | 0.435^***^ | 0.738^***^ | 0.760^***^ | 2.991^***^ | 3.032^***^ | 1.611^***^ | 2.120^***^ | 2.168^***^ |
|  | (0.133) | (0.134) | (0.145) | (0.180) | (0.181) | (0.140) | (0.141) | (0.153) | (0.191) | (0.192) |
| female | 0.027 | 0.028 | 0.011 | 0.012 | 0.012 | 0.133^***^ | 0.131^***^ | 0.116^***^ | 0.118^***^ | 0.115^***^ |
|  | (0.029) | (0.029) | (0.028) | (0.028) | (0.028) | (0.038) | (0.038) | (0.037) | (0.037) | (0.037) |
| age | 0.095^**^ | 0.096^**^ | 0.094^**^ | 0.095^**^ | 0.095^**^ | 0.246^***^ | 0.243^***^ | 0.235^***^ | 0.236^***^ | 0.233^***^ |
|  | (0.047) | (0.047) | (0.045) | (0.045) | (0.045) | (0.060) | (0.060) | (0.059) | (0.059) | (0.059) |
| escs_scale | 0.103^***^ | 0.103^***^ | 0.103^***^ | 0.103^***^ | 0.103^***^ | 0.312^***^ | 0.312^***^ | 0.308^***^ | 0.308^***^ | 0.308^***^ |
|  | (0.019) | (0.019) | (0.018) | (0.018) | (0.018) | (0.021) | (0.021) | (0.021) | (0.021) | (0.021) |
| reading_fluency | -0.005^***^ | -0.005^***^ | -0.006^***^ | -0.006^***^ | -0.006^***^ | -0.011^***^ | -0.011^***^ | -0.011^***^ | -0.011^***^ | -0.011^***^ |
|  | (0.001) | (0.001) | (0.0005) | (0.0005) | (0.0005) | (0.001) | (0.001) | (0.001) | (0.001) | (0.001) |
| ICT_excess_use_cat1high | -0.119^***^ | -0.175^***^ | -0.112^***^ | -0.112^***^ | -0.173^***^ | -0.337^***^ | -0.451^***^ | -0.324^***^ | -0.322^***^ | -0.453^***^ |
|  | (0.029) | (0.046) | (0.028) | (0.028) | (0.045) | (0.036) | (0.055) | (0.035) | (0.035) | (0.055) |
| ICT_excess_use_cat1low | -0.379^***^ | -0.274^**^ | -0.364^***^ | -0.365^***^ | -0.287^***^ | -0.633^***^ | -0.412^***^ | -0.610^***^ | -0.610^***^ | -0.431^***^ |
|  | (0.068) | (0.112) | (0.066) | (0.066) | (0.110) | (0.096) | (0.151) | (0.093) | (0.093) | (0.150) |
| percentile_rank_round | -1.137^***^ | -1.178^***^ | -0.952^***^ | -1.490^***^ | -1.534^***^ | -0.878^***^ | -0.962^***^ | -0.790^***^ | -1.745^***^ | -1.841^***^ |
|  | (0.059) | (0.069) | (0.056) | (0.215) | (0.218) | (0.061) | (0.071) | (0.060) | (0.231) | (0.234) |
| timing_decile |  |  | 0.274^***^ | 0.218^***^ | 0.219^***^ |  |  | 0.243^***^ | 0.150^***^ | 0.150^***^ |
|  |  |  | (0.011) | (0.022) | (0.022) |  |  | (0.011) | (0.024) | (0.024) |
| timing_squ_scale |  |  | -0.018^***^ | -0.017^***^ | -0.017^***^ |  |  | -0.018^***^ | -0.013^***^ | -0.013^***^ |
|  |  |  | (0.001) | (0.002) | (0.002) |  |  | (0.001) | (0.002) | (0.002) |
| ICT_excess_use_cat1high:percentile_rank_round |  | 0.115 |  |  | 0.122^*^ |  | 0.233^***^ |  |  | 0.263^***^ |
|  |  | (0.076) |  |  | (0.073) |  | (0.085) |  |  | (0.084) |
| ICT_excess_use_cat1low:percentile_rank_round |  | -0.214 |  |  | -0.156 |  | -0.431^*^ |  |  | -0.341 |
|  |  | (0.183) |  |  | (0.178) |  | (0.230) |  |  | (0.226) |
| percentile_rank_round:timing_decile |  |  |  | 0.098^***^ | 0.097^**^ |  |  |  | 0.175^***^ | 0.174^***^ |
|  |  |  |  | (0.038) | (0.038) |  |  |  | (0.041) | (0.041) |
| percentile_rank_round:timing_squ_scale |  |  |  | -0.002 | -0.002 |  |  |  | -0.009^**^ | -0.009^**^ |
|  |  |  |  | (0.003) | (0.003) |  |  |  | (0.004) | (0.004) |
|  |  | | | | |  | | | | |
| question FE | Yes | Yes | Yes | Yes | Yes | Yes | Yes | Yes | Yes | Yes |
|  |  | | | | |  | | | | |
| Observations | 153.518 | 153.518 | 152.152 | 152.152 | 152.152 | 131.024 | 131.024 | 129.929 | 129.929 | 129.929 |
| Log Likelihood | -78,558.420 | -78,556.100 | -77,419.040 | -77,379.160 | -77,376.970 | -66,005.270 | -65,998.510 | -65,132.780 | -65,090.970 | -65,083.690 |
| Akaike Inf. Crit. | 157,622.800 | 157,622.200 | 155,348.100 | 155,272.300 | 155,271.900 | 132,516.500 | 132,507.000 | 130,775.600 | 130,695.900 | 130,685.400 |
| Bayesian Inf. Crit. | 160,138.100 | 160,157.300 | 157,880.900 | 157,825.000 | 157,844.500 | 134,991.700 | 135,001.700 | 133,268.100 | 133,208.000 | 133,217.000 |
|  |  |  |  |  |  |  | | | | |

|  | ISL | ISL | ISL | ISL | ISL | ISR | ISR | ISR | ISR | ISR |
| --- | --- | --- | --- | --- | --- | --- | --- | --- | --- | --- |
| Constant | 2.674^***^ | 2.661^***^ | 0.868^***^ | 1.508^***^ | 1.502^***^ | 1.900^***^ | 1.920^***^ | 0.092 | 0.778^***^ | 0.804^***^ |
|  | (0.187) | (0.189) | (0.204) | (0.253) | (0.254) | (0.151) | (0.152) | (0.165) | (0.208) | (0.209) |
| female | 0.328^***^ | 0.328^***^ | 0.297^***^ | 0.299^***^ | 0.299^***^ | 0.325^***^ | 0.326^***^ | 0.292^***^ | 0.293^***^ | 0.294^***^ |
|  | (0.051) | (0.051) | (0.047) | (0.047) | (0.047) | (0.042) | (0.042) | (0.039) | (0.039) | (0.039) |
| age | 0.116 | 0.112 | 0.123 | 0.125 | 0.122 | 0.219^***^ | 0.220^***^ | 0.211^***^ | 0.210^***^ | 0.211^***^ |
|  | (0.088) | (0.088) | (0.082) | (0.082) | (0.082) | (0.069) | (0.069) | (0.065) | (0.065) | (0.065) |
| escs_scale | 0.311^***^ | 0.311^***^ | 0.296^***^ | 0.295^***^ | 0.295^***^ | 0.250^***^ | 0.249^***^ | 0.252^***^ | 0.251^***^ | 0.250^***^ |
|  | (0.034) | (0.034) | (0.032) | (0.032) | (0.032) | (0.026) | (0.026) | (0.025) | (0.025) | (0.025) |
| reading_fluency | -0.003^***^ | -0.003^***^ | -0.004^***^ | -0.004^***^ | -0.004^***^ | -0.005^***^ | -0.006^***^ | -0.006^***^ | -0.006^***^ | -0.006^***^ |
|  | (0.001) | (0.001) | (0.001) | (0.001) | (0.001) | (0.001) | (0.001) | (0.001) | (0.001) | (0.001) |
| ICT_excess_use_cat1high | -0.270^***^ | -0.249^***^ | -0.239^***^ | -0.240^***^ | -0.250^***^ | -0.163^***^ | -0.233^***^ | -0.157^***^ | -0.157^***^ | -0.241^***^ |
|  | (0.052) | (0.073) | (0.049) | (0.049) | (0.072) | (0.042) | (0.062) | (0.039) | (0.039) | (0.060) |
| ICT_excess_use_cat1low | -0.728^***^ | -0.219 | -0.695^***^ | -0.698^***^ | -0.174 | -0.435^***^ | -0.318^***^ | -0.418^***^ | -0.419^***^ | -0.311^***^ |
|  | (0.197) | (0.273) | (0.185) | (0.185) | (0.267) | (0.073) | (0.108) | (0.069) | (0.069) | (0.105) |
| percentile_rank_round | -1.113^***^ | -1.073^***^ | -0.886^***^ | -2.106^***^ | -2.084^***^ | -1.616^***^ | -1.655^***^ | -1.358^***^ | -2.630^***^ | -2.678^***^ |
|  | (0.089) | (0.102) | (0.084) | (0.308) | (0.312) | (0.073) | (0.085) | (0.066) | (0.253) | (0.257) |
| timing_decile |  |  | 0.309^***^ | 0.195^***^ | 0.196^***^ |  |  | 0.295^***^ | 0.173^***^ | 0.173^***^ |
|  |  |  | (0.015) | (0.031) | (0.031) |  |  | (0.012) | (0.026) | (0.026) |
| timing_squ_scale |  |  | -0.020^***^ | -0.013^***^ | -0.013^***^ |  |  | -0.020^***^ | -0.013^***^ | -0.013^***^ |
|  |  |  | (0.001) | (0.003) | (0.003) |  |  | (0.001) | (0.002) | (0.002) |
| ICT_excess_use_cat1high:percentile_rank_round |  | -0.047 |  |  | 0.021 |  | 0.148 |  |  | 0.169^*^ |
|  |  | (0.117) |  |  | (0.113) |  | (0.096) |  |  | (0.092) |
| ICT_excess_use_cat1low:percentile_rank_round |  | -1.245^***^ |  |  | -1.207^***^ |  | -0.255 |  |  | -0.226 |
|  |  | (0.463) |  |  | (0.444) |  | (0.168) |  |  | (0.161) |
| percentile_rank_round:timing_decile |  |  |  | 0.221^***^ | 0.219^***^ |  |  |  | 0.229^***^ | 0.229^***^ |
|  |  |  |  | (0.054) | (0.054) |  |  |  | (0.044) | (0.044) |
| percentile_rank_round:timing_squ_scale |  |  |  | -0.013^***^ | -0.013^***^ |  |  |  | -0.012^***^ | -0.012^***^ |
|  |  |  |  | (0.005) | (0.005) |  |  |  | (0.004) | (0.004) |
|  |  | | | | |  | | | | |
| question FE | Yes | Yes | Yes | Yes | Yes | Yes | Yes | Yes | Yes | Yes |
|  |  | | | | |  | | | | |
| Observations | 79.095 | 79.095 | 78.33 | 78.33 | 78.33 | 123.375 | 123.375 | 122.241 | 122.241 | 122.241 |
| Log Likelihood | -41,499.620 | -41,495.790 | -40,692.590 | -40,669.750 | -40,665.760 | -62,944.120 | -62,940.930 | -61,929.950 | -61,877.920 | -61,874.300 |
| Akaike Inf. Crit. | 83,505.250 | 83,501.590 | 81,895.180 | 81,853.510 | 81,849.530 | 126,394.200 | 126,391.900 | 124,369.900 | 124,269.800 | 124,266.600 |
| Bayesian Inf. Crit. | 85,852.680 | 85,867.580 | 84,258.690 | 84,235.560 | 84,250.120 | 128,854.200 | 128,871.200 | 126,846.900 | 126,766.300 | 126,782.500 |
|  |  |  |  |  |  |  |  |  |  |  |

|  | ITA | ITA | ITA | ITA | ITA | JPN | JPN | JPN | JPN | JPN |
| --- | --- | --- | --- | --- | --- | --- | --- | --- | --- | --- |
| Constant | 2.639^***^ | 2.662^***^ | 0.726^***^ | 1.300^***^ | 1.326^***^ | 1.954^***^ | 1.968^***^ | -0.194 | 0.391^**^ | 0.414^**^ |
|  | (0.098) | (0.099) | (0.107) | (0.132) | (0.133) | (0.123) | (0.124) | (0.134) | (0.167) | (0.167) |
| female | 0.172^***^ | 0.172^***^ | 0.151^***^ | 0.152^***^ | 0.152^***^ | 0.156^***^ | 0.156^***^ | 0.126^***^ | 0.127^***^ | 0.127^***^ |
|  | (0.024) | (0.024) | (0.023) | (0.023) | (0.023) | (0.030) | (0.030) | (0.029) | (0.028) | (0.028) |
| age | 0.187^***^ | 0.187^***^ | 0.187^***^ | 0.185^***^ | 0.185^***^ | -0.039 | -0.039 | -0.026 | -0.025 | -0.026 |
|  | (0.038) | (0.038) | (0.036) | (0.036) | (0.036) | (0.049) | (0.049) | (0.046) | (0.046) | (0.046) |
| escs_scale | 0.090^***^ | 0.090^***^ | 0.091^***^ | 0.092^***^ | 0.092^***^ | 0.109^***^ | 0.109^***^ | 0.108^***^ | 0.109^***^ | 0.110^***^ |
|  | (0.014) | (0.014) | (0.013) | (0.013) | (0.013) | (0.022) | (0.022) | (0.020) | (0.020) | (0.020) |
| reading_fluency | -0.006^***^ | -0.006^***^ | -0.006^***^ | -0.006^***^ | -0.006^***^ | -0.007^***^ | -0.007^***^ | -0.007^***^ | -0.007^***^ | -0.007^***^ |
|  | (0.0004) | (0.0004) | (0.0004) | (0.0004) | (0.0004) | (0.001) | (0.001) | (0.0005) | (0.0005) | (0.0005) |
| ICT_excess_use_cat1high | -0.118^***^ | -0.191^***^ | -0.115^***^ | -0.113^***^ | -0.197^***^ | -0.056 | -0.151^**^ | -0.030 | -0.030 | -0.145^**^ |
|  | (0.023) | (0.037) | (0.022) | (0.022) | (0.036) | (0.039) | (0.061) | (0.036) | (0.036) | (0.059) |
| ICT_excess_use_cat1low | -0.405^***^ | -0.209^***^ | -0.397^***^ | -0.396^***^ | -0.227^***^ | -0.172^***^ | -0.137^**^ | -0.172^***^ | -0.172^***^ | -0.155^**^ |
|  | (0.049) | (0.078) | (0.046) | (0.046) | (0.076) | (0.043) | (0.069) | (0.040) | (0.040) | (0.067) |
| percentile_rank_round | -1.383^***^ | -1.428^***^ | -1.161^***^ | -2.238^***^ | -2.287^***^ | -1.039^***^ | -1.065^***^ | -0.836^***^ | -1.924^***^ | -1.965^***^ |
|  | (0.042) | (0.051) | (0.040) | (0.157) | (0.160) | (0.053) | (0.058) | (0.050) | (0.199) | (0.201) |
| timing_decile |  |  | 0.324^***^ | 0.221^***^ | 0.221^***^ |  |  | 0.365^***^ | 0.258^***^ | 0.258^***^ |
|  |  |  | (0.008) | (0.016) | (0.016) |  |  | (0.010) | (0.020) | (0.020) |
| timing_squ_scale |  |  | -0.022^***^ | -0.018^***^ | -0.018^***^ |  |  | -0.026^***^ | -0.021^***^ | -0.021^***^ |
|  |  |  | (0.001) | (0.001) | (0.001) |  |  | (0.001) | (0.002) | (0.002) |
| ICT_excess_use_cat1high:percentile_rank_round |  | 0.151^**^ |  |  | 0.165^***^ |  | 0.197^**^ |  |  | 0.228^**^ |
|  |  | (0.060) |  |  | (0.057) |  | (0.097) |  |  | (0.092) |
| ICT_excess_use_cat1low:percentile_rank_round |  | -0.409^***^ |  |  | -0.342^***^ |  | -0.072 |  |  | -0.035 |
|  |  | (0.128) |  |  | (0.122) |  | (0.111) |  |  | (0.106) |
| percentile_rank_round:timing_decile |  |  |  | 0.195^***^ | 0.194^***^ |  |  |  | 0.199^***^ | 0.200^***^ |
|  |  |  |  | (0.028) | (0.028) |  |  |  | (0.035) | (0.035) |
| percentile_rank_round:timing_squ_scale |  |  |  | -0.007^***^ | -0.007^***^ |  |  |  | -0.007^**^ | -0.007^**^ |
|  |  |  |  | (0.002) | (0.002) |  |  |  | (0.003) | (0.003) |
|  |  | | | | |  | | | | |
| question FE | Yes | Yes | Yes | Yes | Yes | Yes | Yes | Yes | Yes | Yes |
|  |  | | | | |  | | | | |
| Observations | 297.484 | 297.484 | 295.099 | 295.099 | 295.099 | 180.235 | 180.235 | 178.73 | 178.73 | 178.73 |
| Log Likelihood | -154,588.300 | -154,577.300 | -151,852.300 | -151,666.100 | -151,655.300 | -93,336.420 | -93,333.750 | -91,490.230 | -91,369.300 | -91,365.860 |
| Akaike Inf. Crit. | 309,682.700 | 309,664.600 | 304,214.500 | 303,846.200 | 303,828.600 | 187,178.800 | 187,177.500 | 183,490.500 | 183,252.600 | 183,249.700 |
| Bayesian Inf. Crit. | 312,365.300 | 312,368.400 | 306,916.300 | 306,569.100 | 306,572.700 | 189,734.700 | 189,753.500 | 186,064.300 | 185,846.700 | 185,864.000 |
|  |  |  |  |  |  |  |  |  |  |  |

|  | KOR | KOR | KOR | KOR | KOR | LTU | LTU | LTU | LTU | LTU |
| --- | --- | --- | --- | --- | --- | --- | --- | --- | --- | --- |
| Constant | 2.334^***^ | 2.324^***^ | 0.440^***^ | 0.744^***^ | 0.736^***^ | 1.728^***^ | 1.760^***^ | 0.124 | 0.790^***^ | 0.826^***^ |
|  | (0.128) | (0.129) | (0.138) | (0.168) | (0.168) | (0.107) | (0.108) | (0.118) | (0.152) | (0.153) |
| female | 0.217^***^ | 0.216^***^ | 0.184^***^ | 0.186^***^ | 0.185^***^ | 0.260^***^ | 0.260^***^ | 0.223^***^ | 0.223^***^ | 0.223^***^ |
|  | (0.039) | (0.039) | (0.037) | (0.037) | (0.037) | (0.027) | (0.027) | (0.026) | (0.026) | (0.026) |
| age | 0.016 | 0.014 | 0.024 | 0.024 | 0.023 | 0.122^**^ | 0.123^**^ | 0.117^**^ | 0.116^**^ | 0.117^**^ |
|  | (0.061) | (0.061) | (0.058) | (0.058) | (0.058) | (0.048) | (0.048) | (0.046) | (0.046) | (0.046) |
| escs_scale | 0.243^***^ | 0.243^***^ | 0.240^***^ | 0.239^***^ | 0.239^***^ | 0.232^***^ | 0.233^***^ | 0.230^***^ | 0.230^***^ | 0.230^***^ |
|  | (0.022) | (0.022) | (0.021) | (0.021) | (0.021) | (0.018) | (0.018) | (0.017) | (0.017) | (0.017) |
| reading_fluency | -0.002^***^ | -0.002^***^ | -0.002^***^ | -0.002^***^ | -0.002^***^ | -0.009^***^ | -0.009^***^ | -0.009^***^ | -0.009^***^ | -0.009^***^ |
|  | (0.001) | (0.001) | (0.001) | (0.001) | (0.001) | (0.0005) | (0.0005) | (0.0005) | (0.0005) | (0.0005) |
| ICT_excess_use_cat1high | -0.206^***^ | -0.226^***^ | -0.199^***^ | -0.198^***^ | -0.235^***^ | -0.094^***^ | -0.165^***^ | -0.088^***^ | -0.087^***^ | -0.165^***^ |
|  | (0.042) | (0.061) | (0.040) | (0.040) | (0.060) | (0.028) | (0.044) | (0.027) | (0.027) | (0.043) |
| ICT_excess_use_cat1low | -0.164^***^ | -0.085 | -0.156^***^ | -0.156^***^ | -0.084 | -0.710^***^ | -0.666^***^ | -0.702^***^ | -0.701^***^ | -0.675^***^ |
|  | (0.042) | (0.061) | (0.040) | (0.040) | (0.061) | (0.063) | (0.098) | (0.060) | (0.060) | (0.095) |
| percentile_rank_round | -0.704^***^ | -0.683^***^ | -0.625^***^ | -1.185^***^ | -1.170^***^ | -1.140^***^ | -1.203^***^ | -0.934^***^ | -2.181^***^ | -2.252^***^ |
|  | (0.057) | (0.063) | (0.055) | (0.192) | (0.194) | (0.051) | (0.062) | (0.049) | (0.191) | (0.194) |
| timing_decile |  |  | 0.339^***^ | 0.284^***^ | 0.284^***^ |  |  | 0.271^***^ | 0.151^***^ | 0.151^***^ |
|  |  |  | (0.009) | (0.020) | (0.020) |  |  | (0.009) | (0.020) | (0.020) |
| timing_squ_scale |  |  | -0.026^***^ | -0.024^***^ | -0.024^***^ |  |  | -0.018^***^ | -0.010^***^ | -0.010^***^ |
|  |  |  | (0.001) | (0.002) | (0.002) |  |  | (0.001) | (0.002) | (0.002) |
| ICT_excess_use_cat1high:percentile_rank_round |  | 0.046 |  |  | 0.076 |  | 0.145^**^ |  |  | 0.155^**^ |
|  |  | (0.096) |  |  | (0.094) |  | (0.070) |  |  | (0.067) |
| ICT_excess_use_cat1low:percentile_rank_round |  | -0.172^*^ |  |  | -0.151 |  | -0.091 |  |  | -0.053 |
|  |  | (0.097) |  |  | (0.095) |  | (0.154) |  |  | (0.149) |
| percentile_rank_round:timing_decile |  |  |  | 0.102^***^ | 0.101^***^ |  |  |  | 0.227^***^ | 0.227^***^ |
|  |  |  |  | (0.034) | (0.034) |  |  |  | (0.033) | (0.033) |
| percentile_rank_round:timing_squ_scale |  |  |  | -0.003 | -0.003 |  |  |  | -0.015^***^ | -0.015^***^ |
|  |  |  |  | (0.003) | (0.003) |  |  |  | (0.003) | (0.003) |
|  |  | | | | |  | | | | |
| question FE | Yes | Yes | Yes | Yes | Yes | Yes | Yes | Yes | Yes | Yes |
|  |  | | | | |  | | | | |
| Observations | 211.24 | 211.24 | 209.356 | 209.356 | 209.356 | 192.818 | 192.818 | 191.033 | 191.033 | 191.033 |
| Log Likelihood | -102,214.700 | -102,212.700 | -100,521.600 | -100,484.200 | -100,482.200 | -102,313.800 | -102,310.800 | -100,724.800 | -100,680.400 | -100,677.100 |
| Akaike Inf. Crit. | 204,935.500 | 204,935.500 | 201,553.100 | 201,482.400 | 201,482.400 | 205,133.500 | 205,131.700 | 201,959.700 | 201,874.700 | 201,872.200 |
| Bayesian Inf. Crit. | 207,531.400 | 207,552.000 | 204,167.300 | 204,117.100 | 204,137.600 | 207,706.400 | 207,724.900 | 204,550.500 | 204,485.900 | 204,503.700 |
|  |  |  |  |  |  |  |  |  |  |  |

|  | LVA | LVA | LVA | LVA | LVA | MEX | MEX | MEX | MEX | MEX |
| --- | --- | --- | --- | --- | --- | --- | --- | --- | --- | --- |
| Constant | 2.365^***^ | 2.415^***^ | 0.863^***^ | 1.508^***^ | 1.558^***^ | 2.309^***^ | 2.347^***^ | 1.131^***^ | 1.453^***^ | 1.497^***^ |
|  | (0.124) | (0.126) | (0.137) | (0.176) | (0.177) | -0.11 | -0.11 | -0.12 | -0.15 | -0.15 |
| female | 0.315^***^ | 0.315^***^ | 0.283^***^ | 0.284^***^ | 0.284^***^ | 0.076^***^ | 0.077^***^ | 0.060^**^ | 0.060^**^ | 0.061^**^ |
|  | (0.032) | (0.032) | (0.031) | (0.031) | (0.031) | -0.03 | -0.03 | -0.02 | -0.02 | -0.02 |
| age | 0.103^*^ | 0.102^*^ | 0.105^*^ | 0.105^**^ | 0.105^**^ | 0.124^***^ | 0.122^***^ | 0.124^***^ | 0.124^***^ | 0.122^***^ |
|  | (0.056) | (0.056) | (0.054) | (0.053) | (0.053) | -0.04 | -0.04 | -0.04 | -0.04 | -0.04 |
| escs_scale | 0.228^***^ | 0.228^***^ | 0.227^***^ | 0.226^***^ | 0.226^***^ | 0.075^***^ | 0.076^***^ | 0.082^***^ | 0.082^***^ | 0.083^***^ |
|  | (0.021) | (0.021) | (0.021) | (0.021) | (0.021) | -0.01 | -0.01 | -0.01 | -0.01 | -0.01 |
| reading_fluency | -0.007^***^ | -0.007^***^ | -0.008^***^ | -0.008^***^ | -0.008^***^ | -0.008^***^ | -0.008^***^ | -0.008^***^ | -0.008^***^ | -0.008^***^ |
|  | (0.001) | (0.001) | (0.001) | (0.001) | (0.001) | 0.00 | 0.00 | 0.00 | 0.00 | 0.00 |
| ICT_excess_use_cat1high | -0.148^***^ | -0.254^***^ | -0.139^***^ | -0.139^***^ | -0.245^***^ | -0.01 | -0.122^***^ | 0.00 | 0.00 | -0.116^***^ |
|  | (0.033) | (0.051) | (0.032) | (0.032) | (0.050) | -0.03 | -0.05 | -0.03 | -0.03 | -0.05 |
| ICT_excess_use_cat1low | -0.618^***^ | -0.695^***^ | -0.608^***^ | -0.611^***^ | -0.682^***^ | -0.342^***^ | -0.294^***^ | -0.335^***^ | -0.334^***^ | -0.298^***^ |
|  | (0.081) | (0.126) | (0.078) | (0.078) | (0.123) | -0.04 | -0.06 | -0.04 | -0.04 | -0.06 |
| percentile_rank_round | -1.096^***^ | -1.204^***^ | -0.916^***^ | -2.121^***^ | -2.224^***^ | -1.097^***^ | -1.167^***^ | -0.920^***^ | -1.488^***^ | -1.568^***^ |
|  | (0.058) | (0.070) | (0.056) | (0.221) | (0.224) | -0.05 | -0.06 | -0.05 | -0.19 | -0.20 |
| timing_decile |  |  | 0.259^***^ | 0.141^***^ | 0.142^***^ |  |  | 0.198^***^ | 0.141^***^ | 0.140^***^ |
|  |  |  | (0.011) | (0.023) | (0.023) |  |  | -0.01 | -0.02 | -0.02 |
| timing_squ_scale |  |  | -0.018^***^ | -0.011^***^ | -0.011^***^ |  |  | -0.012^***^ | -0.010^***^ | -0.010^***^ |
|  |  |  | (0.001) | (0.002) | (0.002) |  |  | 0.00 | 0.00 | 0.00 |
| ICT_excess_use_cat1high:percentile_rank_round |  | 0.226^***^ |  |  | 0.219^***^ |  | 0.218^***^ |  |  | 0.225^***^ |
|  |  | (0.083) |  |  | (0.080) |  | -0.07 |  |  | -0.07 |
| ICT_excess_use_cat1low:percentile_rank_round |  | 0.164 |  |  | 0.149 |  | -0.09 |  |  | -0.07 |
|  |  | (0.203) |  |  | (0.196) |  | -0.09 |  |  | -0.09 |
| percentile_rank_round:timing_decile |  |  |  | 0.219^***^ | 0.219^***^ |  |  |  | 0.103^***^ | 0.103^***^ |
|  |  |  |  | (0.039) | (0.039) |  |  |  | -0.03 | -0.03 |
| percentile_rank_round:timing_squ_scale |  |  |  | -0.013^***^ | -0.013^***^ |  |  |  | 0.00 | 0.00 |
|  |  |  |  | (0.003) | (0.003) |  |  |  | 0.00 | 0.00 |
|  |  | | | | |  | | | | |
| question FE | Yes | Yes | Yes | Yes | Yes | Yes | Yes | Yes | Yes | Yes |
|  |  | | | | |  | | | | |
| Observations | 142.079 | 142.079 | 140.726 | 140.726 | 140.726 | 174,422 | 174,422 | 172,779 | 172,779 | 172,779 |
| Log Likelihood | -76,168.440 | -76,164.530 | -75,056.070 | -75,016.290 | -75,012.350 | -96,693.39 | -96,685.98 | -95,265.05 | -95,234.26 | -95,226.82 |
| Akaike Inf. Crit. | 152,842.900 | 152,839.100 | 150,622.100 | 150,546.600 | 150,542.700 | 193,892.80 | 193,882.00 | 191,040.10 | 190,982.50 | 190,971.60 |
| Bayesian Inf. Crit. | 155,338.500 | 155,354.400 | 153,135.100 | 153,079.200 | 153,095.000 | 196,440.30 | 196,449.60 | 193,605.30 | 193,567.90 | 193,577.10 |
|  |  |  |  |  |  |  |  |  |  |  |

|  | NZL | NZL | NZL | NZL | NZL | POL | POL | POL | POL | POL |
| --- | --- | --- | --- | --- | --- | --- | --- | --- | --- | --- |
| Constant | 2.857^***^ | 2.826^***^ | 1.086^***^ | 1.675^***^ | 1.645^***^ | 2.536^***^ | 2.560^***^ | 0.757^***^ | 1.217^***^ | 1.239^***^ |
|  | -0.15 | -0.15 | -0.17 | -0.21 | -0.21 | -0.13 | -0.13 | -0.14 | -0.17 | -0.17 |
| female | 0.197^***^ | 0.198^***^ | 0.168^***^ | 0.172^***^ | 0.173^***^ | 0.268^***^ | 0.269^***^ | 0.234^***^ | 0.235^***^ | 0.235^***^ |
|  | -0.04 | -0.04 | -0.04 | -0.04 | -0.04 | -0.03 | -0.03 | -0.03 | -0.03 | -0.03 |
| age | 0.223^***^ | 0.223^***^ | 0.218^***^ | 0.217^***^ | 0.218^***^ | 0.08 | 0.08 | 0.08 | 0.09 | 0.09 |
|  | -0.07 | -0.07 | -0.07 | -0.07 | -0.07 | -0.06 | -0.06 | -0.05 | -0.05 | -0.05 |
| escs_scale | 0.362^***^ | 0.364^***^ | 0.356^***^ | 0.356^***^ | 0.358^***^ | 0.348^***^ | 0.348^***^ | 0.346^***^ | 0.345^***^ | 0.345^***^ |
|  | -0.02 | -0.02 | -0.02 | -0.02 | -0.02 | -0.02 | -0.02 | -0.02 | -0.02 | -0.02 |
| reading_fluency | -0.010^***^ | -0.010^***^ | -0.010^***^ | -0.010^***^ | -0.010^***^ | -0.008^***^ | -0.008^***^ | -0.009^***^ | -0.009^***^ | -0.009^***^ |
|  | 0.00 | 0.00 | 0.00 | 0.00 | 0.00 | 0.00 | 0.00 | 0.00 | 0.00 | 0.00 |
| ICT_excess_use_cat1high | -0.289^***^ | -0.238^***^ | -0.276^***^ | -0.277^***^ | -0.230^***^ | -0.134^***^ | -0.199^***^ | -0.123^***^ | -0.121^***^ | -0.185^***^ |
|  | -0.04 | -0.06 | -0.04 | -0.04 | -0.06 | -0.03 | -0.05 | -0.03 | -0.03 | -0.05 |
| ICT_excess_use_cat1low | -0.465^***^ | -0.24 | -0.449^***^ | -0.454^***^ | -0.20 | -0.498^***^ | -0.370^***^ | -0.485^***^ | -0.483^***^ | -0.373^***^ |
|  | -0.11 | -0.16 | -0.11 | -0.11 | -0.16 | -0.09 | -0.13 | -0.08 | -0.08 | -0.13 |
| percentile_rank_round | -1.100^***^ | -1.030^***^ | -0.970^***^ | -2.075^***^ | -2.011^***^ | -1.249^***^ | -1.300^***^ | -1.047^***^ | -1.904^***^ | -1.950^***^ |
|  | -0.07 | -0.08 | -0.07 | -0.25 | -0.25 | -0.06 | -0.07 | -0.06 | -0.21 | -0.21 |
| timing_decile |  |  | 0.308^***^ | 0.203^***^ | 0.203^***^ |  |  | 0.304^***^ | 0.220^***^ | 0.220^***^ |
|  |  |  | -0.01 | -0.03 | -0.03 |  |  | -0.01 | -0.02 | -0.02 |
| timing_squ_scale |  |  | -0.023^***^ | -0.017^***^ | -0.017^***^ |  |  | -0.021^***^ | -0.017^***^ | -0.017^***^ |
|  |  |  | 0.00 | 0.00 | 0.00 |  |  | 0.00 | 0.00 | 0.00 |
| ICT_excess_use_cat1high:percentile_rank_round |  | -0.11 |  |  | -0.10 |  | 0.139^*^ |  |  | 0.131^*^ |
|  |  | -0.09 |  |  | -0.09 |  | -0.08 |  |  | -0.08 |
| ICT_excess_use_cat1low:percentile_rank_round |  | -0.459^*^ |  |  | -0.501^**^ |  | -0.26 |  |  | -0.22 |
|  |  | -0.25 |  |  | -0.24 |  | -0.21 |  |  | -0.21 |
| percentile_rank_round:timing_decile |  |  |  | 0.200^***^ | 0.200^***^ |  |  |  | 0.156^***^ | 0.156^***^ |
|  |  |  |  | -0.04 | -0.04 |  |  |  | -0.04 | -0.04 |
| percentile_rank_round:timing_squ_scale |  |  |  | -0.011^***^ | -0.011^***^ |  |  |  | -0.007^**^ | -0.007^**^ |
|  |  |  |  | 0.00 | 0.00 |  |  |  | 0.00 | 0.00 |
|  |  | | | | |  | | | | |
| question FE | Yes | Yes | Yes | Yes | Yes | Yes | Yes | Yes | Yes | Yes |
|  |  | | | | |  | | | | |
| Observations | 124,949 | 124,949 | 123,900 | 123,900 | 123,900 | 166,437 | 166,437 | 165,008 | 165,008 | 165,008 |
| Log Likelihood | -63,206.27 | -63,204.00 | -62,292.68 | -62,257.60 | -62,255.04 | -84,170.64 | -84,167.95 | -82,837.16 | -82,793.76 | -82,791.38 |
| Akaike Inf. Crit. | 126,918.50 | 126,918.00 | 125,095.40 | 125,029.20 | 125,028.10 | 168,847.30 | 168,845.90 | 166,184.30 | 166,101.50 | 166,100.80 |
| Bayesian Inf. Crit. | 129,381.70 | 129,400.60 | 127,575.80 | 127,529.10 | 127,547.40 | 171,382.90 | 171,401.60 | 168,737.80 | 168,675.00 | 168,694.30 |
|  |  |  |  |  |  |  |  |  |  |  |

|  | SVK | SVK | SVK | SVK | SVK | SVN | SVN | SVN | SVN | SVN |
| --- | --- | --- | --- | --- | --- | --- | --- | --- | --- | --- |
| Constant | 2.487^***^ | 2.488^***^ | 0.704^***^ | 1.436^***^ | 1.434^***^ | 2.789^***^ | 2.808^***^ | 1.322^***^ | 1.850^***^ | 1.868^***^ |
|  | -0.12 | -0.12 | -0.13 | -0.17 | -0.17 | -0.14 | -0.14 | -0.15 | -0.18 | -0.18 |
| female | 0.196^***^ | 0.196^***^ | 0.175^***^ | 0.177^***^ | 0.177^***^ | 0.200^***^ | 0.200^***^ | 0.177^***^ | 0.179^***^ | 0.178^***^ |
|  | -0.03 | -0.03 | -0.03 | -0.03 | -0.03 | -0.03 | -0.03 | -0.03 | -0.03 | -0.03 |
| age | 0.01 | 0.01 | 0.00 | 0.00 | 0.00 | 0.100^**^ | 0.100^**^ | 0.092^**^ | 0.092^**^ | 0.093^**^ |
|  | -0.06 | -0.06 | -0.06 | -0.06 | -0.06 | -0.05 | -0.05 | -0.05 | -0.05 | -0.05 |
| escs_scale | 0.230^***^ | 0.230^***^ | 0.227^***^ | 0.226^***^ | 0.226^***^ | 0.066^***^ | 0.067^***^ | 0.071^***^ | 0.071^***^ | 0.071^***^ |
|  | -0.02 | -0.02 | -0.02 | -0.02 | -0.02 | -0.02 | -0.02 | -0.02 | -0.02 | -0.02 |
| reading_fluency | -0.007^***^ | -0.008^***^ | -0.007^***^ | -0.007^***^ | -0.007^***^ | -0.006^***^ | -0.006^***^ | -0.006^***^ | -0.006^***^ | -0.006^***^ |
|  | 0.00 | 0.00 | 0.00 | 0.00 | 0.00 | 0.00 | 0.00 | 0.00 | 0.00 | 0.00 |
| ICT_excess_use_cat1high | -0.120^***^ | -0.146^***^ | -0.114^***^ | -0.114^***^ | -0.130^***^ | -0.141^***^ | -0.212^***^ | -0.126^***^ | -0.126^***^ | -0.194^***^ |
|  | -0.03 | -0.05 | -0.03 | -0.03 | -0.05 | -0.03 | -0.05 | -0.03 | -0.03 | -0.05 |
| ICT_excess_use_cat1low | -0.396^***^ | -0.253^***^ | -0.386^***^ | -0.385^***^ | -0.276^***^ | -0.342^***^ | -0.275^***^ | -0.334^***^ | -0.333^***^ | -0.271^***^ |
|  | -0.06 | -0.10 | -0.06 | -0.06 | -0.09 | -0.06 | -0.09 | -0.05 | -0.05 | -0.09 |
| percentile_rank_round | -1.516^***^ | -1.517^***^ | -1.207^***^ | -2.581^***^ | -2.574^***^ | -1.036^***^ | -1.074^***^ | -0.860^***^ | -1.824^***^ | -1.859^***^ |
|  | -0.06 | -0.07 | -0.05 | -0.21 | -0.21 | -0.05 | -0.06 | -0.05 | -0.20 | -0.21 |
| timing_decile |  |  | 0.291^***^ | 0.160^***^ | 0.160^***^ |  |  | 0.253^***^ | 0.157^***^ | 0.157^***^ |
|  |  |  | -0.01 | -0.02 | -0.02 |  |  | -0.01 | -0.02 | -0.02 |
| timing_squ_scale |  |  | -0.018^***^ | -0.011^***^ | -0.011^***^ |  |  | -0.017^***^ | -0.012^***^ | -0.012^***^ |
|  |  |  | 0.00 | 0.00 | 0.00 |  |  | 0.00 | 0.00 | 0.00 |
| ICT_excess_use_cat1high:percentile_rank_round |  | 0.05 |  |  | 0.03 |  | 0.150^*^ |  |  | 0.141^*^ |
|  |  | -0.08 |  |  | -0.08 |  | -0.08 |  |  | -0.07 |
| ICT_excess_use_cat1low:percentile_rank_round |  | -0.296^*^ |  |  | -0.22 |  | -0.15 |  |  | -0.13 |
|  |  | -0.15 |  |  | -0.15 |  | -0.15 |  |  | -0.14 |
| percentile_rank_round:timing_decile |  |  |  | 0.248^***^ | 0.247^***^ |  |  |  | 0.176^***^ | 0.175^***^ |
|  |  |  |  | -0.04 | -0.04 |  |  |  | -0.04 | -0.04 |
| percentile_rank_round:timing_squ_scale |  |  |  | -0.013^***^ | -0.013^***^ |  |  |  | -0.009^***^ | -0.009^***^ |
|  |  |  |  | 0.00 | 0.00 |  |  |  | 0.00 | 0.00 |
|  |  | | | | |  | | | | |
| question FE | Yes | Yes | Yes | Yes | Yes | Yes | Yes | Yes | Yes | Yes |
|  |  | | | | |  | | | | |
| Observations | 167,089 | 167,089 | 165,504 | 165,504 | 165,504 | 172,174 | 172,174 | 170,656 | 170,656 | 170,656 |
| Log Likelihood | -87,887.42 | -87,884.86 | -86,245.65 | -86,158.06 | -86,156.64 | -88,494.99 | -88,492.04 | -87,228.24 | -87,181.51 | -87,178.82 |
| Akaike Inf. Crit. | 176,280.80 | 176,279.70 | 173,001.30 | 172,830.10 | 172,831.30 | 177,496.00 | 177,494.10 | 174,966.50 | 174,877.00 | 174,875.60 |
| Bayesian Inf. Crit. | 178,817.50 | 178,836.40 | 175,555.60 | 175,404.40 | 175,425.60 | 180,040.20 | 180,058.40 | 177,528.60 | 177,459.20 | 177,477.90 |
|  |  |  |  |  |  |  |  |  |  |  |

|  | SWE | SWE | SWE | SWE | SWE | TUR | TUR | TUR | TUR | TUR |
| --- | --- | --- | --- | --- | --- | --- | --- | --- | --- | --- |
| Constant | 3.233^***^ | 3.281^***^ | 0.923^***^ | 1.537^***^ | 1.600^***^ | 1.407^***^ | 1.415^***^ | 0.370^***^ | 0.903^***^ | 0.908^***^ |
|  | -0.17 | -0.18 | -0.19 | -0.23 | -0.24 | -0.11 | -0.11 | -0.12 | -0.15 | -0.15 |
| female | 0.247^***^ | 0.248^***^ | 0.218^***^ | 0.218^***^ | 0.219^***^ | 0.091^***^ | 0.091^***^ | 0.088^***^ | 0.088^***^ | 0.088^***^ |
|  | -0.04 | -0.04 | -0.04 | -0.04 | -0.04 | -0.02 | -0.02 | -0.02 | -0.02 | -0.02 |
| age | 0.254^***^ | 0.253^***^ | 0.243^***^ | 0.240^***^ | 0.239^***^ | 0.01 | 0.00 | 0.00 | 0.00 | 0.00 |
|  | -0.07 | -0.07 | -0.07 | -0.07 | -0.07 | -0.04 | -0.04 | -0.04 | -0.04 | -0.04 |
| escs_scale | 0.332^***^ | 0.332^***^ | 0.323^***^ | 0.322^***^ | 0.322^***^ | 0.043^***^ | 0.043^***^ | 0.043^***^ | 0.043^***^ | 0.043^***^ |
|  | -0.03 | -0.03 | -0.03 | -0.03 | -0.03 | -0.01 | -0.01 | -0.01 | -0.01 | -0.01 |
| reading_fluency | -0.010^***^ | -0.010^***^ | -0.010^***^ | -0.010^***^ | -0.010^***^ | -0.003^***^ | -0.003^***^ | -0.003^***^ | -0.003^***^ | -0.003^***^ |
|  | 0.00 | 0.00 | 0.00 | 0.00 | 0.00 | 0.00 | 0.00 | 0.00 | 0.00 | 0.00 |
| ICT_excess_use_cat1high | -0.348^***^ | -0.432^***^ | -0.320^***^ | -0.319^***^ | -0.421^***^ | -0.03 | -0.079^*^ | -0.03 | -0.02 | -0.078^*^ |
|  | -0.04 | -0.06 | -0.04 | -0.04 | -0.06 | -0.02 | -0.04 | -0.02 | -0.02 | -0.04 |
| ICT_excess_use_cat1low | -1.577^***^ | -1.874^***^ | -1.437^***^ | -1.423^***^ | -1.762^***^ | -0.210^***^ | -0.158^***^ | -0.201^***^ | -0.200^***^ | -0.154^***^ |
|  | -0.23 | -0.36 | -0.22 | -0.22 | -0.35 | -0.03 | -0.05 | -0.03 | -0.03 | -0.05 |
| percentile_rank_round | -1.418^***^ | -1.526^***^ | -1.222^***^ | -2.422^***^ | -2.556^***^ | -0.845^***^ | -0.859^***^ | -0.778^***^ | -1.818^***^ | -1.826^***^ |
|  | -0.07 | -0.10 | -0.07 | -0.28 | -0.28 | -0.05 | -0.05 | -0.04 | -0.18 | -0.18 |
| timing_decile |  |  | 0.392^***^ | 0.281^***^ | 0.281^***^ |  |  | 0.181^***^ | 0.084^***^ | 0.085^***^ |
|  |  |  | -0.01 | -0.03 | -0.03 |  |  | -0.01 | -0.02 | -0.02 |
| timing_squ_scale |  |  | -0.029^***^ | -0.023^***^ | -0.023^***^ |  |  | -0.012^***^ | -0.007^***^ | -0.007^***^ |
|  |  |  | 0.00 | 0.00 | 0.00 |  |  | 0.00 | 0.00 | 0.00 |
| ICT_excess_use_cat1high:percentile_rank_round |  | 0.181^*^ |  |  | 0.210^**^ |  | 0.10 |  |  | 0.11 |
|  |  | -0.10 |  |  | -0.10 |  | -0.07 |  |  | -0.07 |
| ICT_excess_use_cat1low:percentile_rank_round |  | 0.58 |  |  | 0.64 |  | -0.11 |  |  | -0.10 |
|  |  | -0.54 |  |  | -0.51 |  | -0.09 |  |  | -0.09 |
| percentile_rank_round:timing_decile |  |  |  | 0.216^***^ | 0.218^***^ |  |  |  | 0.190^***^ | 0.188^***^ |
|  |  |  |  | -0.05 | -0.05 |  |  |  | -0.03 | -0.03 |
| percentile_rank_round:timing_squ_scale |  |  |  | -0.012^***^ | -0.012^***^ |  |  |  | -0.010^***^ | -0.010^***^ |
|  |  |  |  | 0.00 | 0.00 |  |  |  | 0.00 | 0.00 |
|  |  | | | | |  | | | | |
| question FE | Yes | Yes | Yes | Yes | Yes | Yes | Yes | Yes | Yes | Yes |
|  |  | | | | |  | | | | |
| Observations | 111,376 | 111,376 | 110,461 | 110,461 | 110,461 | 197,362 | 197,362 | 195,648 | 195,648 | 195,648 |
| Log Likelihood | -55,101.52 | -55,099.50 | -54,174.63 | -54,140.58 | -54,137.69 | -104,219.50 | -104,216.90 | -102,909.30 | -102,838.70 | -102,836.10 |
| Akaike Inf. Crit. | 110,707.00 | 110,707.00 | 108,857.30 | 108,793.20 | 108,791.40 | 208,943.00 | 208,941.80 | 206,326.60 | 206,189.50 | 206,188.20 |
| Bayesian Inf. Crit. | 113,131.40 | 113,150.60 | 111,298.80 | 111,253.90 | 111,271.40 | 211,511.60 | 211,530.70 | 208,913.30 | 208,796.60 | 208,815.60 |
|  |  |  |  |  |  |  |  |  |  |  |

|  | USA | USA | USA | USA | USA |
| --- | --- | --- | --- | --- | --- |
| Constant | 2.789^***^ | 2.793^***^ | 1.124^***^ | 1.744^***^ | 1.753^***^ |
|  | -0.159 | -0.161 | -0.174 | -0.217 | -0.219 |
| female | 0.161^***^ | 0.160^***^ | 0.139^***^ | 0.141^***^ | 0.140^***^ |
|  | -0.043 | -0.043 | -0.042 | -0.042 | -0.042 |
| age | 0.242^***^ | 0.240^***^ | 0.234^***^ | 0.233^***^ | 0.232^***^ |
|  | -0.075 | -0.075 | -0.072 | -0.072 | -0.072 |
| escs_scale | 0.313^***^ | 0.313^***^ | 0.306^***^ | 0.306^***^ | 0.306^***^ |
|  | -0.026 | -0.026 | -0.025 | -0.025 | -0.025 |
| reading_fluency | -0.012^***^ | -0.012^***^ | -0.012^***^ | -0.012^***^ | -0.012^***^ |
|  | -0.001 | -0.001 | -0.001 | -0.001 | -0.001 |
| ICT_excess_use_cat1high | -0.222^***^ | -0.243^***^ | -0.217^***^ | -0.216^***^ | -0.243^***^ |
|  | -0.045 | -0.065 | -0.043 | -0.043 | -0.064 |
| ICT_excess_use_cat1low | -0.456^***^ | -0.218 | -0.447^***^ | -0.450^***^ | -0.229 |
|  | -0.111 | -0.16 | -0.106 | -0.106 | -0.158 |
| percentile_rank_round | -0.998^***^ | -1.000^***^ | -0.870^***^ | -1.976^***^ | -1.986^***^ |
|  | -0.072 | -0.089 | -0.07 | -0.256 | -0.261 |
| timing_decile |  |  | 0.288^***^ | 0.176^***^ | 0.176^***^ |
|  |  |  | -0.013 | -0.027 | -0.027 |
| timing_squ_scale |  |  | -0.022^***^ | -0.015^***^ | -0.015^***^ |
|  |  |  | -0.001 | -0.002 | -0.002 |
| ICT_excess_use_cat1high:percentile_rank_round |  | 0.043 |  |  | 0.056 |
|  |  | -0.098 |  |  | -0.095 |
| ICT_excess_use_cat1low:percentile_rank_round |  | -0.500^**^ |  |  | -0.450^*^ |
|  |  | -0.244 |  |  | -0.237 |
| percentile_rank_round:timing_decile |  |  |  | 0.202^***^ | 0.202^***^ |
|  |  |  |  | -0.045 | -0.045 |
| percentile_rank_round:timing_squ_scale |  |  |  | -0.012^***^ | -0.012^***^ |
|  |  |  |  | -0.004 | -0.004 |
|  |  | | | | |
| question FE | Yes | Yes | Yes | Yes | Yes |
|  |  | | | | |
| Observations | 109,557 | 109,557 | 108,618 | 108,618 | 108,618 |
| Log Likelihood | -55,739.88 | -55,737.32 | -54,938.88 | -54,907.20 | -54,904.82 |
| Akaike Inf. Crit. | 111,985.80 | 111,984.60 | 110,387.80 | 110,328.40 | 110,327.60 |
| Bayesian Inf. Crit. | 114,415.60 | 114,433.70 | 112,834.60 | 112,794.50 | 112,812.90 |
|  |  |  |  |  |  |
